# Preterm birth, infant weight gain, and childhood asthma risk: A meta-analysis of 147,000 European children

**DOI:** 10.1016/j.jaci.2013.12.1082

**Published:** 2014-05

**Authors:** Agnes M.M. Sonnenschein-van der Voort, Lidia R. Arends, Johan C. de Jongste, Isabella Annesi-Maesano, S. Hasan Arshad, Henrique Barros, Mikel Basterrechea, Hans Bisgaard, Leda Chatzi, Eva Corpeleijn, Sofia Correia, Leone C. Craig, Graham Devereux, Cristian Dogaru, Miroslav Dostal, Karel Duchen, Merete Eggesbø, C. Kors van der Ent, Maria P. Fantini, Francesco Forastiere, Urs Frey, Ulrike Gehring, Davide Gori, Anne C. van der Gugten, Wojciech Hanke, A. John Henderson, Barbara Heude, Carmen Iñiguez, Hazel M. Inskip, Thomas Keil, Cecily C. Kelleher, Manolis Kogevinas, Eskil Kreiner-Møller, Claudia E. Kuehni, Leanne K. Küpers, Kinga Lancz, Pernille S. Larsen, Susanne Lau, Johnny Ludvigsson, Monique Mommers, Anne-Marie Nybo Andersen, Lubica Palkovicova, Katharine C. Pike, Costanza Pizzi, Kinga Polanska, Daniela Porta, Lorenzo Richiardi, Graham Roberts, Anne Schmidt, Radim J. Sram, Jordi Sunyer, Carel Thijs, Maties Torrent, Karien Viljoen, Alet H. Wijga, Martine Vrijheid, Vincent W.V. Jaddoe, Liesbeth Duijts

**Affiliations:** a Generation R Study Group, Erasmus Medical Center, Rotterdam, The Netherlands; b Department of Epidemiology, Erasmus Medical Center, Rotterdam, The Netherlands; c Department of Paediatrics, Division of Respiratory Medicine, Erasmus Medical Center, Rotterdam, The Netherlands; d Department of Biostatistics, Erasmus Medical Center, Rotterdam, The Netherlands; xx Department of Paediatrics, Erasmus Medical Center, Rotterdam, The Netherlands; yy Department of Paediatrics, Division of Neonatology, Erasmus Medical Center, Rotterdam, The Netherlands; e Institute of Pedagogical Sciences, Erasmus University Rotterdam, Rotterdam, The Netherlands; f Institute of Psychology, Erasmus University Rotterdam, Rotterdam, The Netherlands; g EPAR, UMR-S 707 INSERM Paris, Paris, France; h EPAR, UMR-S 707, Université Pierre et Marie Curie Paris 06, Paris, France; i David Hide Asthma and Allergy Research Centre, St Mary's Hospital, Newport, Isle of Wight, United Kingdom; j Department of Clinical Epidemiology, Predictive Medicine and Public Health, University of Porto Medical School, Porto, Portugal; k Public Health Division of Gipuzkoa, Gipuzkoa, Spain; l Spanish Consortium for Research on Epidemiology and Public Health (CIBERESP), Barcelona, Spain; m Copenhagen Prospective Studies on Asthma in Childhood (COPSAC), Faculty of Health Sciences, University of Copenhagen, Copenhagen, Denmark; mm Section of Social Medicine, Department of Public Health, University of Copenhagen, Copenhagen, Denmark; n Danish Pediatric Asthma Center, Copenhagen University Hospital, Gentofte, Denmark; o Department of Social Medicine, School of Medicine, University of Crete, Crete, Greece; p Department of Epidemiology, University of Groningen, University Medical Center Groningen, Groningen, The Netherlands; q Applied Health Sciences, University of Aberdeen, Aberdeen, United Kingdom; r Institute of Social and Preventive Medicine, University of Bern, Bern, Switzerland; s Institute of Experimental Medicine, Academy of Sciences of the Czech Republic, Prague, Czech Republic; t Division of Pediatrics, Department of Clinical and Experimental Medicine, Linköping University, and Pediatric Clinic, County Council of Östergötland County Council, Linköping, Sweden; u Department of Genes and Environment, Division of Epidemiology, Norwegian Institute of Public Health, Oslo, Norway; v Department of Paediatric Pulmonology, Wilhelmina Children's Hospital, University Medical Center Utrecht, Utrecht, The Netherlands; w Department of Biomedical and Neuromotor Sciences, University of Bologna, Bologna, Italy; x Department of Epidemiology, Lazio Regional Health Service, Rome, Italy; y University Children's Hospital Basel (UKBB), University of Basel, Basel, Switzerland; z Institute for Risk Assessment Sciences, Utrecht University, Utrecht, The Netherlands; aa Nofer Institute of Occupational Medicine, Department of Environmental Epidemiology, Lodz, Poland; bb School of Social and Community Medicine, University of Bristol, Bristol, United Kingdom; cc INSERM, Center for Research in Epidemiology and Population Health, U1018, Lifelong Epidemiology Of Obesity, Diabetes, and Renal Disease Team, Villejuif, France; dd University Paris-Sud, Villejuif, France; ee Center for Public Health Research (CSISP), University of Valencia, Valencia, Spain; ff Faculty of nursery and chiropody, University of Valencia, Valencia, Spain; gg MRC Lifecourse Epidemiology Unit, University of Southampton, Southampton General Hospital, Southampton, United Kingdom; hh Institute of Social Medicine, Epidemiology and Health Economics, Charité University Medical Center, Berlin, Germany; ii Institute for Clinical Epidemiology and Biometry, University of Würzburg, Würzburg, Germany; jj School of Public Health, Physiotherapy and Population Science, University College Dublin, Dublin, Ireland; kk National School of Public Health, Athens, Greece; ll Department of Environmental Medicine, Faculty of Public Health, Slovak Medical University, Bratislava, Slovakia; nn Department of Paediatric Pneumology and Immunology, Charité University Medical Centre, Berlin, Germany; oo Department of Epidemiology, CAPHRI School for Public Health and Primary Care, Maastricht University, Maastricht, The Netherlands; pp Clinical and Experimental Sciences Academic Unit, Faculty of Medicine, University of Southampton, Southampton, United Kingdom; qq Cancer Epidemiology Unit, Department of Medical Sciences, University of Turin, Turin, Italy; rr Division of Respiratory Medicine, Department of Pediatrics, Inselspital, University of Bern, Bern, Switzerland; ss Centre for Research in Environmental Epidemiology (CREAL), Barcelona, Spain; tt Department of Experimental and Health Sciences, Pompeu Fabra University, Barcelona, Spain; uu Institut Municipal d’Investigació Mèdica (IMIM)–Hospital del Mar, Barcelona, Spain; vv IB-SALUT, Area de Salut de Menorca, Balearic Islands, Spain; ww Centre for Prevention and Health Services Research, National Institute for Public Health and the Environment (RIVM), Bilthoven, The Netherlands

**Keywords:** Gestational age, low birth weight, infant growth, wheezing, asthma, children, cohort studies, epidemiology, BMI, Body mass index, ISAAC, International Study on Asthma and Allergy in Childhood, OR, Odds ratio, pOR, Pooled odds ratio, SDS, Standard deviation scores

## Abstract

**Background:**

Preterm birth, low birth weight, and infant catch-up growth seem associated with an increased risk of respiratory diseases in later life, but individual studies showed conflicting results.

**Objectives:**

We performed an individual participant data meta-analysis for 147,252 children of 31 birth cohort studies to determine the associations of birth and infant growth characteristics with the risks of preschool wheezing (1-4 years) and school-age asthma (5-10 years).

**Methods:**

First, we performed an adjusted 1-stage random-effect meta-analysis to assess the combined associations of gestational age, birth weight, and infant weight gain with childhood asthma. Second, we performed an adjusted 2-stage random-effect meta-analysis to assess the associations of preterm birth (gestational age <37 weeks) and low birth weight (<2500 g) with childhood asthma outcomes.

**Results:**

Younger gestational age at birth and higher infant weight gain were independently associated with higher risks of preschool wheezing and school-age asthma (*P* < .05). The inverse associations of birth weight with childhood asthma were explained by gestational age at birth. Compared with term-born children with normal infant weight gain, we observed the highest risks of school-age asthma in children born preterm with high infant weight gain (odds ratio [OR], 4.47; 95% CI, 2.58-7.76). Preterm birth was positively associated with an increased risk of preschool wheezing (pooled odds ratio [pOR], 1.34; 95% CI, 1.25-1.43) and school-age asthma (pOR, 1.40; 95% CI, 1.18-1.67) independent of birth weight. Weaker effect estimates were observed for the associations of low birth weight adjusted for gestational age at birth with preschool wheezing (pOR, 1.10; 95% CI, 1.00-1.21) and school-age asthma (pOR, 1.13; 95% CI, 1.01-1.27).

**Conclusion:**

Younger gestational age at birth and higher infant weight gain were associated with childhood asthma outcomes. The associations of lower birth weight with childhood asthma were largely explained by gestational age at birth.

Respiratory diseases have at least part of their origins in early life. It has been hypothesized that adverse exposures in fetal and early postnatal life might influence lung growth and development, which could lead to persistently smaller airways and impaired lung function. These developmental adaptations might predispose the subject to asthma and chronic obstructive pulmonary disease in childhood and adulthood.^1-3^ This hypothesis is supported by studies showing associations of low birth weight with an increased risk of wheezing and asthma in childhood^4-7^ and chronic obstructive pulmonary disease and lower pulmonary function in later life.^8-11^ Published findings are not consistent,^4-7,12,13^ which might be due to differences in study populations and in definitions of outcomes. Also, the observed associations of low birth weight with an increased risk of asthma-related outcomes might be confounded by preterm birth or catch-up growth in infancy. The lungs of preterm children have not yet been fully developed, which makes them prone to suboptimal further development.^14-16^

Most children with low birth weight show catch-up growth in infancy.^17^ Recent studies suggested that catch-up growth is associated with lower pulmonary function and an increased risk of childhood asthma.^18-20^ Whether and to what extent the previously reported associations of low birth weight with higher risks of asthma-related outcomes are explained by preterm birth and infant catch-up growth is not known.

Therefore we conducted a meta-analysis of individual data from 147,252 children up to the age of 10 years participating in 31 European cohort studies to assess the strength, consistency, and independence of the associations of gestational age, birth weight, and infant weight gain with the risk of preschool wheezing and school-age asthma. We specifically explored the combined effects of gestational age, birth weight, and infant growth.

## Methods

### Inclusion criteria and participating cohorts

**Fig E1:**
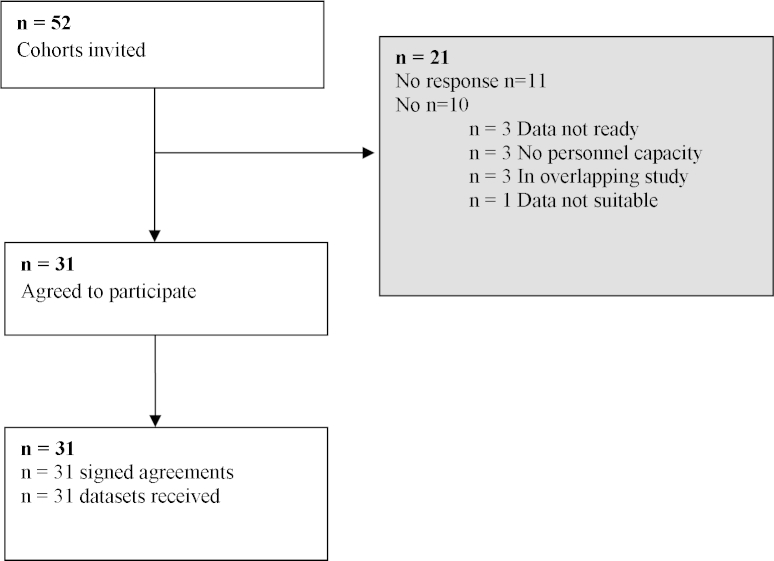


European population-based birth and mother-child cohorts participated if they included children born between 1989 and 2011, had information available on at least gestational age and weight at birth and preschool wheezing (1-4 years) or school-age asthma (5-10 years), and were willing and able to exchange original data. We identified 52 European cohorts selected from the existing collaborations on childhood health or asthma-related outcomes (www.chicosproject.eu, www.birthcohortsenrieco.net, www.ga2len.org, and www.birthcohorts.net assessed until May 29, 2012). We invited the 52 potentially eligible cohorts, of which 41 responded to our invitation. From those, 31 cohorts agreed to participate, leading to 147,252 children with information on at least 1 early growth characteristic and respiratory outcome (see the flow chart shown in Fig E1 in this article's Online Repository at www.jacionline.org). All original cohort studies were approved by their local institutional review boards and provided written informed consent for using their data. Anonymized data sets were stored on a single central secured data server with access for the main analysts (A.M.M.S., L.R.A., and L.D.) only.

### Birth characteristics and infant growth

Information about birth weight, gestational age at birth, and weight in the first year of life per cohort was obtained by using measurements, medical registries, or parental questionnaires (cohort-specific information is shown in Table E1 in this article's Online Repository at www.jacionline.org) and used as continuous and categorical variables. Infant weight gain in the first year was defined as the difference between weight at 1 year (range, 6-18 months) and weight at birth divided by the exact number of months between those 2 measurements. We created gestational age–adjusted birth weight standard deviation scores (SDS) based on a North-European reference chart.^21^ No general European or World Health Organization reference curves of birth weight for gestational age are available. To test nonlinear and dose-response associations, we categorized gestational age (<28.0, 28.0-29.9, 30.0-31.9, 32.0-33.9, 34.0-35.9, 36.0-37.9, 38.0-39.9, 40-41.9, and ≥42 weeks), birth weight SDS (<−4, −4 to −3.01, −3 to −2.01, −2 to −1.01, −1 to −0.01, 0 to 0.99, 1 to 1.99, 2 to 2.99, 3 to 3.99, and ≥4 SDS), and infant weight gain (<300, 300-399, 400-499, 500-599, 600-699, 700-799, 800-899, 900-999, and ≥1000 g/mo). To test the combined associations of gestational age, birth weight SDS, and infant weight gain with childhood asthma outcomes, we used a smaller number of groups to have sufficient children per group (for gestational age: <32, 32-35.9, 36-39.9, and ≥40 weeks; for birth weight SDS: <−2; −2 to −1.01, −1 to 0.99, 1 to 1.99, and ≥2 SDs; and for infant weight gain: <500, 500-599, 600-699, and ≥700 g/mo). Finally, we dichotomized gestational age at birth into term birth (≥37 weeks) and preterm birth (gestational age, <37 weeks) and birth weight into normal birth weight (≥2500 g) and low birth weight (<2500 g) to test the effects of clinical birth complications on childhood asthma outcomes. Cohort-specific characteristics of determinants are shown in Table E2 in this article's Online Repository at www.jacionline.org.

### Asthma-related outcomes in childhood

We used preschool wheezing and school-age asthma as the main outcomes. These data were mainly obtained by using questionnaires adapted from the International Study on Asthma and Allergy in Childhood (ISAAC).^22^ Cohort-specific information is shown in Table E1. We defined preschool wheezing as “ever reported wheezing during the first 4 years of life (no, yes)” and school-age asthma as “asthma diagnosis reported between 5 and 10 years (no, yes),” preferably physician diagnosed. If cohorts had repeatedly collected data on ever wheezing in the first 4 years or asthma diagnosis between 5 and 10 years of life, we used data collected at the oldest age.

### Covariates

We included covariates based on known associations with childhood asthma from previous studies.^23-27^ Information on covariates was mostly assessed by using questionnaires (see Table E1). The individual cohort analyses were adjusted for potential confounders, including maternal educational level (low, medium, high), smoking during pregnancy (no, yes), history of asthma (no, yes), smoking during infancy of their offspring (no, yes), child's sex (female, male), siblings (no, yes), and attending day care in the first 2 years of lice (no, yes; description of available covariates per cohort is shown in Table E3 in this article's Online Repository at www.jacionline.org). We considered breast-feeding status (never, ever), lower respiratory tract infections (no, yes), and eczema (no, yes) in the first 2 years of life as potential intermediates (description of available intermediates per cohort is shown in Table E4 in this article's Online Repository at www.jacionline.org).

### Statistical analysis

First, we performed a 1-stage random-effect meta-analysis on an individual participant's data to examine the separate and combined associations of gestational age, birth weight, and infant weight gain with preschool wheezing and school-age asthma. For this analysis, individual participants' data from all cohorts were included in 1 multilevel analysis and were analyzed, simultaneously taking into account clustering of participants within studies.^28^ Because we used a Northern European reference curve for birth weight for gestational age (birth weight SDS), we performed a sensitivity analysis to explore whether the association was different in Northwest European subjects only (Denmark, France, Germany, Ireland, The Netherlands, Norway, Sweden, Switzerland, and United Kingdom).^29^ Numbers were too low to perform these analyses separately in other European regions.

Second, we performed a 2-stage random-effect meta-analysis to examine the associations of gestational age at birth, birth weight, and infant weight gain and dichotomized preterm birth and low birth weight with the risks of preschool wheezing and school-age asthma. For this analysis, which was used for the clinically relevant associations of preterm birth and low birth weight, we first used logistic regression models to calculate effect estimates per cohort and then used calculated pooled odds ratios (pORs) from the per-cohort effect estimates.^28^ To enable comparison of effect estimates, results for birth weight and infant weight gain are presented as pORs per 500 g/mo and 100 g/mo increase, respectively, which reflect the corresponding SDs. We tested for heterogeneity by calculating the Cochran *Q* and *I*^*2*^ values, which varied per analysis.^30^ We used random-effects models, which take into account the potential between-study variation next to the within-study variation.^31^ To determine the influence of any particular cohort on overall results, we repeated each meta-analysis, leaving out 1 cohort at the time. The first model was adjusted for the child's sex (crude model), the second model was additionally adjusted for potential confounders (confounder model), and the third model was additionally adjusted for potential intermediates (intermediate model). We considered the confounder model as the main model. Results are presented as forest plots or tables with central point estimates from the random-effect models with their 95% CIs. The number of cohorts and children per meta-analysis differed because of differences in data availability. For all analyses, missing values in covariates were used as an additional group in the categorical variables to prevent exclusion of noncomplete cases. We also performed a complete-case sensitivity analysis to explore any differences with complete-case analyses and sensitivity analyses in which we first excluded children with parent-reported birth weight and then excluded children without ISAAC-based questionnaires on wheezing. Statistical analyses were performed with SAS 9.2 software (SAS Institute, Cary, NC) and Comprehensive Meta-Analysis (Biostat).

## Results

### Subjects' characteristics

The cohort-specific information about the main exposures and outcomes are shown in Table I. The overall prevalences of preterm birth (gestational age <37 weeks) and low birth weight (<2500 g) were 5.1% and 3.9%, respectively. Overall preschool wheezing prevalence was 31.6%, and overall school-age asthma prevalence was 12.8%.

### Gestational age, birth weight, and infant weight gain

**Fig E2:**
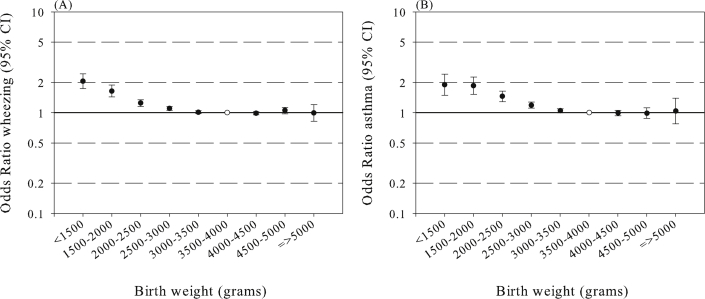


**Fig 1:**
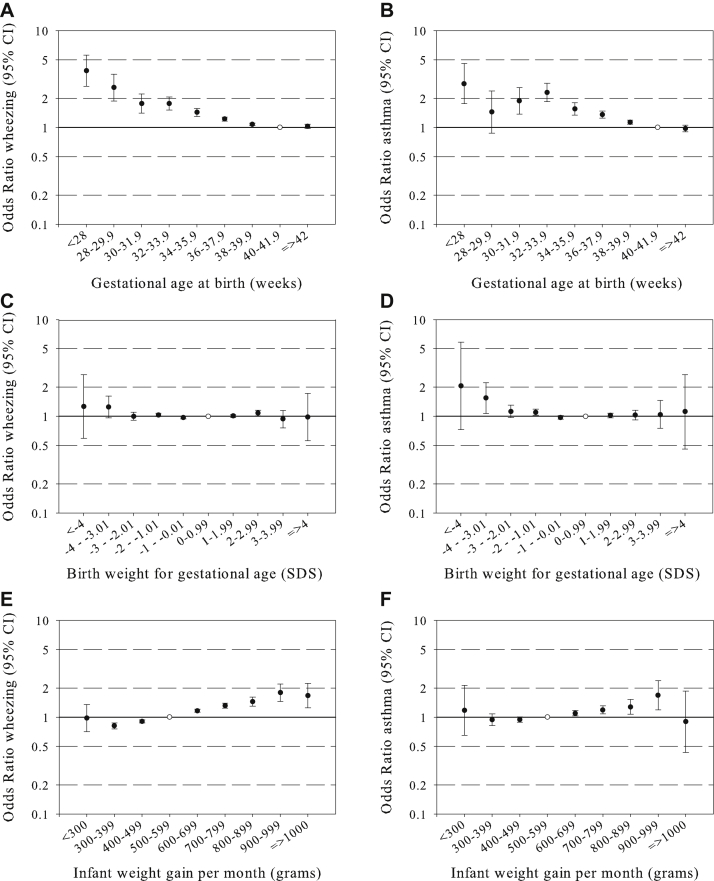
Associations of gestational age at birth, birth weight, and infant weight gain with preschool wheezing and school-age asthma. Values are ORs (95% CIs) from multilevel models for the associations of gestational age at birth (**A** and **B**), gestational age–adjusted birth weight (**C** and **D**), and gestational age–and birth weight–adjusted infant weight gain (**E** and **F**) with asthma outcomes. Models are adjusted for confounders (see the Methods section). Reference groups are represented by *open circles*.

In the 1-stage meta-analysis of individual participants' data, we observed consistent inverse associations of gestational age at birth with the risk of preschool wheezing and school-age asthma. Compared with term-born children, children born before 28 weeks of gestation had the highest risk of preschool wheezing (odds ratio [OR], 3.87; 95% CI, 2.70-5.53) and school-age asthma (OR, 2.92; 95% CI, 1.84-4.62; Fig 1, *A* and *B*). Almost all children born before a gestational age of 40.0 weeks had an increased risk of preschool wheezing and school-age asthma. Birth weight SDS were not consistently associated with childhood asthma outcomes (Fig 1, *C* and *D*). Results for birth weight in grams without taking gestational age into account are shown in Fig E2 in this article's Online Repository at www.jacionline.org, showing an inverse association. We observed a positive association of infant weight gain with preschool wheezing and school-age asthma. Compared with children with a weight gain of between 500 and 600 g/mo (largest group), children with a mean infant weight gain of between 900 and 1000 g/mo had the highest risk of preschool wheezing (OR, 1.79; 95% CI, 1.45-2.21) and school-age asthma (OR, 1.69; 95% CI, 1.19-2.38; Fig 1, *E* and *F*). The overall results for the linear associations of gestational age at birth, birth weight, and infant weight gain from the 1-stage meta-analysis of individual participants' data were similar to those from the 2-stage meta-analysis of individual participants' data (results are shown in Table E5 in this article's Online Repository at www.jacionline.org). The results from the confounder model were not materially different from those from the crude model. Also, additionally adjusting the confounder model for potential intermediates (breast-feeding, lower respiratory tract infections, and eczema) did not materially change the effect estimates (results are shown in Tables E6 and E7 in this article's Online Repository at www.jacionline.org). Also, we observed similar effect estimates for preschool wheezing and school-age asthma after excluding cohorts one by one, indicating no disturbing effect of any particular population (data not shown). After exclusion of the Danish National Birth Cohort, the largest cohort in our meta-analysis (COPSAC) and a high-risk asthma and atopy cohort, we also did not observe major changes in effect estimates (data not shown).

**Fig 2:**
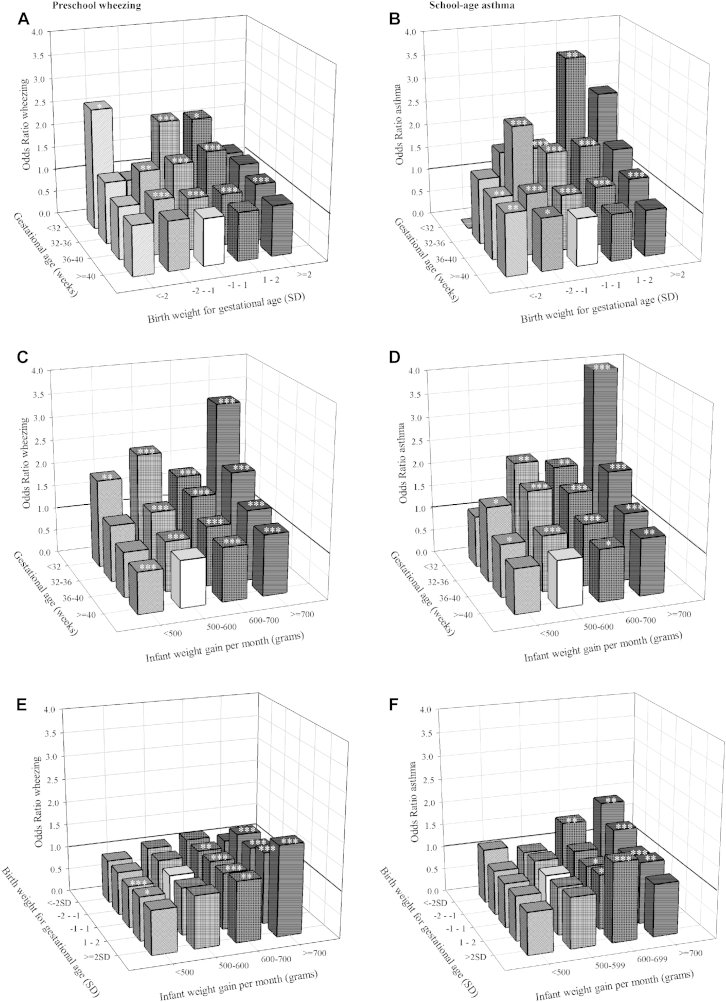
Combined associations of gestational age at birth, birth weight, and infant weight gain with preschool wheezing and school-age asthma. Values are ORs (95% CIs) from multilevel models for the associations of gestational age at birth and birth weight SDSs (**A** and **B**), gestational age at birth and infant weight gain (**C** and **D**), and birth weight SDSs and infant weight gain (**E** and **F**) with asthma outcomes. Models are adjusted for confounders (see the Methods section). Reference groups are represented by a *white bar*. *P* values for gestational age*SD birth weight interactions are as follows: wheezing, .97; asthma, .04. *P* values for gestational age*weight gain interaction are as follows: wheezing, .05; asthma, .23. *P* values for birth weight SDS*weight gain interactions are as follows: wheezing, .15; asthma, .57. *P < .05, **P < .01, and ***P < .001.

Next, we explored the combined effects of gestational age at birth, birth weight SDS, and infant weight gain. The significant correlations were between gestational age and birth weight (*r* = 0.58, *P* < .001), between gestational age and infant weight gain (*r* = −0.16, *P* < .001), and between birth weight and infant weight gain (*r* = −0.12, *P* < .001). We performed stratified analyses and an overall test for interaction. In each analysis the largest group was used as the reference group. For the combined effect analysis of gestational age at birth and birth weight SDS, we observed a higher risk of preschool wheezing among children born at an earlier age with higher birth weight SDS, but the overall interaction term with birth weight SDS was not significant (Fig 2, *A*). Similarly, we observed a tendency toward a higher risk of school-age asthma in children born at an earlier gestational age with higher birth weight SDS (*P* for interaction = .04; Fig 2, *B*). The highest risks for school-age asthma were observed for children born before 32 weeks of gestation with a moderately high birth weight SDS (OR, 3.47; 95% CI, 1.65-7.31) and with a high birth weight SDS (OR, 2.63; 95% CI, 0.53-13.13) compared with children born at term with a normal birth weight SDS. The *P* value for interaction between gestational age at birth and infant weight gain for the associations with preschool wheezing and school-age asthma were 0.05 and 0.23, respectively (Fig 2, *C* and *D*). We observed the highest risk of preschool wheezing and school-age asthma among children born before 32 weeks of gestation with an infant weight gain of more than 700 g compared with children born at term with a normal weight gain (ORs of 3.27 [95% CI, 2.06-5.19] and 4.47 [95% CI, 2.58-7.76], respectively). The interactions between birth weight SDSs and infant weight gain for the associations with preschool wheezing and school-age wheezing were not significant (Fig 2, *E* and *F*). As a sensitivity analysis, we performed our analysis in Northwest European cohorts only and observed similar results (results are shown in Tables E8 and E9 in this article's Online Repository at www.jacionline.org). The results of complete case analyses were similar (data not shown). Also, we observed similar effect estimates for preschool wheezing and school-age asthma after excluding cohorts that used parental reports of birth weight or non–ISAAC-based questions on wheezing. With this exclusion, only the youngest and lowest weight-for-gestational-age groups tended to show stronger effects (see Tables E10 and E11 in this article's Online Repository at www.jacionline.org). This indicates that differences in data collection did not lead to systematic differences in effect estimates.

### Preterm birth, low birth weight, and childhood asthma outcomes

**Fig 3:**
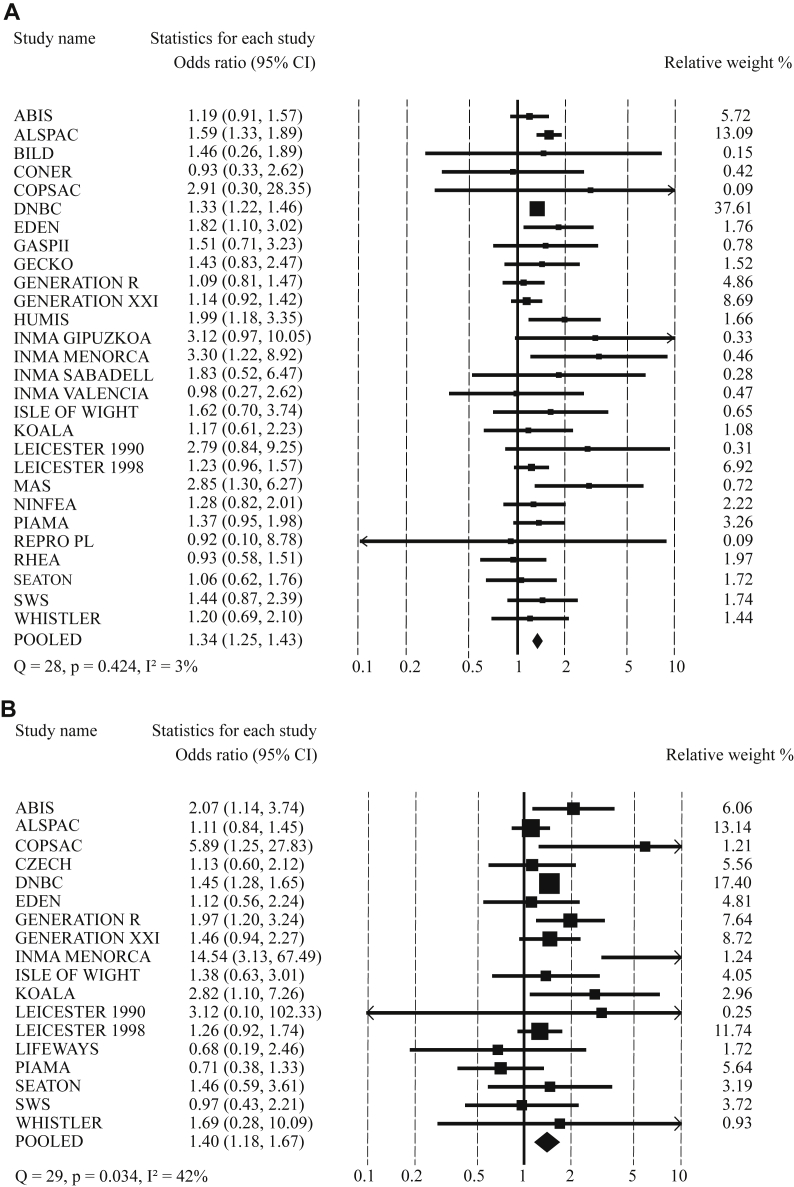

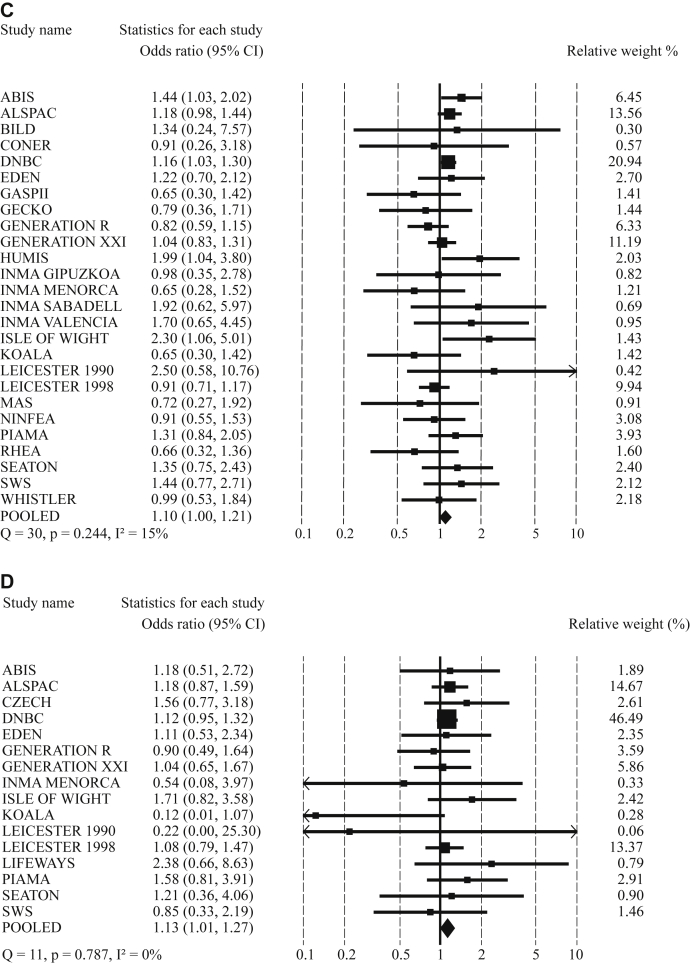
Meta-analysis for associations of preterm birth and low birth weight with preschool wheezing and school-age asthma. **A,** Preterm birth and preschool wheezing. **B,** Preterm birth and school-age asthma. **C,** Low birth weight and preschool wheezing. **D,** Low birth weight and school-age asthma. Values from random-effects models reflect ORs (95% CIs) of preschool wheezing and school-age asthma in preterm children (<37 weeks) compared with those in children born at term (≥37 weeks) adjusted for birth weight (**A** and **B**) and of preschool wheezing and school-age asthma in low-birth-weight children (<2500 g) compared with children born with a normal birth weight (≥2500 g) adjusted for gestational age at birth (**C** and **D**). *Arrows* represent 95% CIs that exceed the outer limits (0.1-10). Models are adjusted for confounders (see the Methods section).

Results from the 2-stage meta-analysis focused on the associations of preterm birth and low birth weight with childhood asthma outcomes are shown in Fig 3. Compared with term-born children, preterm-born children had an increased risk of preschool wheezing (pOR, 1.34; 95% CI, 1.25-1.43) and school-age asthma (pOR, 1.40; 95% CI, 1.18-1.67; Fig 3, *A* and *B*). These associations were independent of birth weight. The population-attributable risk of preterm birth was 1.96% for preschool wheezing and 2.14% for school-age asthma. Compared with children with a normal birth weight, those with a low birth weight (<2500 g) had an increased risk of preschool wheezing (pOR, 1.10; 95% CI, 1.00-1.21) and school-age asthma (pOR, 1.13; 95% CI, 1.01-1.27; Fig 3, *C* and *D*). These associations were stronger without adjustment for gestational age at birth (results are shown in Table E6).

## Discussion

Results from this large-scale meta-analysis of individual participants' data suggested that younger gestational age at birth and higher infant weight gain were associated with increased risks of preschool wheezing and school-age asthma. The associations of low birth weight with childhood asthma outcomes were largely explained by gestational age at birth. The highest risk for childhood asthma outcomes was observed among children born before a gestational age of 32 weeks with high infant weight gain.

### Comparison with earlier studies

Adverse exposures in fetal and early postnatal life can lead to developmental lung adaptations, such as persistently smaller airways and impaired lung function. These developmental adaptations might predispose the patients to obstructive pulmonary diseases in childhood and adulthood.^1-3^ This hypothesis is supported by studies showing associations of low birth weight with an increased risk of wheezing and asthma in childhood.^4-11^ Because low birth weight is correlated with gestational age at birth and infant weight gain, we aimed to disentangle the associations of both gestational age at birth, gestational age–adjusted birth weight, and infant weight gain with childhood asthma outcomes.

Jaakkola et al^16^ performed a meta-analysis on the associations of preterm birth with asthma based on 19 published cohort, case-control, and cross-sectional studies. They observed that preterm-born children, which were defined as those born before 37 weeks of gestation, had an increased risk of asthma between 1 and 24 years of age, with a similar effect estimate as observed in our group of 5- to 10-year-olds. They did not assess associations of birth weight with asthma outcomes. Also, Flaherman and Rutherford^32^ performed a meta-analysis on 12 previously published prospective and retrospective studies and suggested that children with a high weight at birth had an increased risk of asthma between 6 months and 31 years of age. They were not able to explore the role of confounders or the effect of gestational age at birth. No association of gestational age with childhood asthma was presented. Because these reports were based on published results, they might be biased and unable to take account of differences in adjustment. A recent analysis by Rzehak et al^33^ of 8 European cohort studies with 12,050 participants observed an increased incidence of asthma until the age of 6 years in children with a high gain of body mass index (BMI) in the first 2 years. In line with this study, we observed increased risk for wheezing and asthma in children with an increased infant weight gain.

Combining childhood asthma outcomes from different age periods is not easy. Asthma is a difficult clinical diagnosis and cannot easily be diagnosed in children younger than 5 years. Many studies used asthma-related outcomes, such as wheezing and shortness of breath, as main outcomes in children. Wheezing seems to be the strongest risk factor for childhood asthma.^34^ Still, wheezing in different age periods might reflect different physiologic mechanisms.^35^ For example, wheezing in infants might reflect viral airway infections instead of asthma.^35^ Therefore we used both wheezing in preschool children and asthma diagnosis in school-age children as outcomes. We observed that both a younger gestational age at birth and higher infant weight gain were associated with an increased risk of preschool wheezing and school-age asthma. For both gestational age at birth and infant weight gain, we observed dose-response associations with childhood asthma outcomes. The associations were not restricted to the extremes of the distribution but present across the full range of gestational age at birth and infant weight gain. To the best of our knowledge, this study is the first showing these associations within the normal ranges. Our results also suggest that the previously observed associations of low birth weight with childhood asthma were largely explained by gestational age at birth. We observed the highest risk of childhood asthma outcomes among children born before a gestational age of 32 weeks with high weight gain in infancy.

### Interpretation of main findings

Mechanisms underlying the associations of factors in early life with asthma outcomes in later childhood might include smaller airways and lungs.^36^ The highest rates of airway and alveolar development occur in early life, and growth and development of the airways and alveoli might continue until the age of 21 years.^37,38^ Extremely premature-born children with respiratory distress syndrome or chronic lung disease commonly have impaired lung function in later life.^39,40^ Follow-up studies in preterm children showed persistently lower lung volumes and reduced airway caliber in later life.^40-44^ However, these extremes do not explain our associations within the less extreme range of gestational age. Children born preterm also have higher chemokine and cytokine levels in nasopharyngeal aspirates at 1 year compared with term-born children, which suggests that preterm-born children are more responsive to proinflammatory stimuli.^45^ The observed associations of high infant weight gain with childhood asthma outcomes are in line with previous studies reporting associations of BMI or adiposity with asthma.^33,46,47^ These associations might be explained by immunologically active factors from adipose tissue, such as leptin.^48^ In mice leptin has been shown to enhance airway hyperresponsiveness, suggesting an immunomodulatory role.^49^ Results in human subjects are inconsistent.^50-52^ High infant weight gain might also have a direct mechanical effect on lung function.^53^ Further studies are needed to identify the developmental adaptations of the lungs and immune system that might explain the associations of preterm birth and infant weight gain with childhood asthma.

### Strengths and limitations

We performed a large meta-analysis of individual participants' data from many birth cohorts throughout Europe. We did not rely on published data, which limits any potential publication bias. The large number of participants enabled us to assess small effects and to adjust for various potential confounders. We presented results from random-effects models, which allow heterogeneity in the true effect estimates between different populations and take between-study variation into account. Another strength is that information on exposures in early life was collected from records and did not depend on long-term participant recall. Misclassification of gestational age is always possible because of the large number of pregnant women who did not know their exact gestational duration.^54,55^ Misclassification of gestational age might have increased the number of children born postterm with a small size for gestational age and children born preterm with a large size for gestational age. Most cohorts used standardized and validated questionnaires to assess wheezing and asthma. This method is widely accepted in epidemiologic studies and reliably reflects the incidence of wheezing and asthma in children.^22,56^ Multiple imputation has been suggested to be the preferable method to deal with missing values.^57^ However, we did not have additional data on patterns of missing values and were therefore unable to perform multiple imputations within cohorts. We used missing values in covariates as an additional group to prevent exclusion of noncomplete cases. No differences in results were observed between the missing as extra category and complete case analyses. In the current study we were not able to assess the effects of early growth characteristics on other objective asthma-related outcomes, such as lung function or bronchial hyperresponsiveness. Although we did take major potential confounders into account, residual confounding might still be an issue. For example, although cohorts comprised predominantly white children, we were unable to adjust for ethnicity. Also, we were unable to adjust for maternal BMI or chorioamnionitis, which might influence growth and inflammatory factors associated with childhood asthma.^58,59^ We were not able to take BMI at the time of obtaining information on childhood asthma outcomes into account. Especially the associations of infant weight gain with childhood asthma outcomes might be explained by later adiposity. Childhood adiposity might be an intermediate in this association.

### Conclusions

Younger gestational age at birth and higher weight gain in infancy were associated with childhood asthma outcomes. The association of lower birth weight with childhood asthma outcomes was largely explained by gestational age at birth. Further studies are needed to evaluate the effects of early-life characteristics on specific asthma-related outcomes, such as lung function, airway size, and airway inflammation.

**Clinical implications**

Children born at a younger gestational age at birth or higher infant weight gain have increased risks of childhood asthma.

## Figures and Tables

**Table I:** Characteristics of the participating European birth cohorts

Cohort name (country)	No. (total = 147,252)	Birth years	Birth weight (g), mean (SD)	Gestational age at birth (wk), median (5% to 95% range)	Preschool wheezing, % (no.)	School-age asthma, % (no.)	Available covariates						
							Maternal education	Prenatal smoke	Maternal asthma	Postnatal smoke	Sex	Siblings	Day care
ABIS (Sweden)	6,829	1997-1998	3,576 (537)	40 (37-42)	32.6 (2,200)	9.9 (258)	√	√		√	√	√	√
ALSPAC (United Kingdom)	12,485	1991-1992	3,403 (554)	40 (36-42)	45.8 (5,683)	21.7 (1,622)	√	√	√	√	√	√	√
BILD (Switzerland)	432	1999	3,382 (441)	39 (37-41)	20.6 (89)	—	√	√	√		√	√	
CONER (Italy)	389	2004-2005	3,321 (448)	39 (37-41)	41.4 (161)	—	√	√	√	√	√	√	
COPSAC (Denmark)	384	1998-2001	3,513 (524)	40 (37-42)	89.5 (331)	18.2 (62)	√	√	√	√	√	√	√
CZECH (Czech Republic)	1,830	2001-2004	3,331 (519)	40 (36-41)	—	13.3 (244)	√	√	√	√	√	√	
DNBC (Denmark)	76,810	1996-2001	3,594 (555)	40 (37-42)	26.9 (17,671)	12.4 (6,498)	√	√	√	√	√	√	√
EDEN (France)	1,774	2003-2005	3,285 (506)	40 (36-41)	32.3 (573)	12.8 (227)	√	√	√	√	√	√	√
GASPII (Italy)	694	2003-2004	3,313 (529)	40 (36-41)	43.7 (303)	—	√	√	√	√	√	√	√
GECKO Drenthe (The Netherlands)	1,718	2006-2007	3,557 (544)	40 (37-42)	29.2 (501)	—	√	√		√	√	√	√
GENERATION R (The Netherlands)	5,815	2002-2006	3,428 (575)	40 (37-42)	29.3 (1,505)	6.0 (263)	√	√	√	√	√	√	√
GENERATION XXI (Portugal)	7,053	2005-2006	3,149 (533)	39 (35-41)	53.0 (2,970)	4.4 (305)	√	√	√		√	√	
HUMIS (Norway)	2,001	2003-2008	3,534 (677)	40 (34-42)	15.0 (301)	—	√	√	√	√	√	√	√
INMA Gipuzkoa (Spain)	478	2006-2008	3,298 (446)	40 (37-42)	35.8 (171)	—	√	√	√	√	√	√	√
INMA Menorca (Spain)	474	1997-1998	3,186 (498)	40 (37-41)	47.9 (227)	6.4 (27)	√	√	√	√	√	√	√
INMA Sabadell (Spain)	502	2004-2007	3,253 (412)	40 (37-42)	59.8 (300)	—	√	√	√	√	√	√	√
INMA Valencia (Spain)	604	2003-2005	3,247 (501)	40 (37-42)	25.7 (155)	—	√	√	√	√	√	√	√
ISLE OF WIGHT (United Kingdom)	1,405	1989-1990	3,411 (523)	40 (38-42)	24.2 (263)	20.1 (272)		√	√		√	√	
KOALA (The Netherlands)	2,151	2000-2003	3,525 (499)	40 (38-42)	24.7 (494)	7.6 (134)	√	√	√		√	√	√
LEICESTER 1990 (United Kingdom)	1,231	1990	3,381 (555)	40 (36-41)	15.0 (156)	30.6 (136)		√			√	√	
LEICESTER 1998 (United Kingdom)	6,836	1998	3,289 (582)	39 (36-41)	38.0 (2,242)	22.3 (1,029)	√	√	√		√	√	
LIFEWAYS (Ireland)	421	2001-2002	3,526 (565)	40 (38-42)	—	26.4 (111)	√	√			√	√	√
MAS (Germany)	1,263	1990	3,412 (463)	40 (37-42)	18.8 (237)	6.6 (44)	√	√	√	√	√	√	√
NINFEA (Italy)	1,922	2005-2010	3,215 (508)	40 (36-42)	23.9 (460)	—	√	√	√	√	√	√	√
PCB (Slovakia)	429	2001-2004	3,359 (492)	40 (38-41)	5.6 (24)	—	√		√		√	√	
PIAMA (The Netherlands)	3,631	1996-1997	3,515 (543)	40 (37-42)	27.3 (964)	10.1 (327)	√	√	√	√	√	√	√
REPRO PL (Poland)	314	2007-2011	3,349 (480)	40 (37-41)	12.4 (39)	—	√	√		√	√	√	√
RHEA (Greece)	1,046	2007-2008	3,179 (437)	38 (36-40)	25.7 (269)	—	√	√	√	√	√	√	√
SEATON (United Kingdom)	1,891	1997	3,414 (610)	40 (35-42)	27.3 (517)	14.7 (131)	√	√	√	√	√	√	√
SWS (United Kingdom)	2,291	1998-2007	3,442 (555)	40 (37-42)	70.9 (1,614)	15.4 (145)	√	√	√	√	√	√	
WHISTLER (The Netherlands)	2,149	2001-2012	3,525 (513)	40 (37-42)	27.2 (577)	7.7 (43)	√	√	√		√	√	√

*No.*, Number of participants with information on at least birth weight or gestational and a respiratory outcome.

## Supplementary data

Tables E1-E11 and Online Figure Legends E1 and E2
