# Supplementary material for: Preterm birth, infant weight gain, and childhood asthma risk: A meta-analysis of 147,000 European children
Source: J Allergy Clin Immunol. 2014 May;133(5):1317–29. doi: 10.1016/j.jaci.2013.12.1082 (PMC4024198; doi:10.1016/j.jaci.2013.12.1082)
Supplement: Tables E1-E11 and Online Figure Legends E1 and E2 [file mmc1.docx]

**Preterm birth, infant weight gain and childhood asthma risk: A meta-analysis of 147,000 European children**

Online-only supplements

Agnes MM Sonnenschein-van der Voort, MSc et al

Table E1 Data collection on early growth characteristics and respiratory outcomes per cohort

Table E2 Characteristics of cohorts: determinants

Table E3 Characteristics of cohorts: confounders

Table E4 Characteristics of cohorts: intermediates

Table E5 Confounder models for the associations of birth weight, gestational age and infant weight gain with preschool wheezing and school-age asthma

Table E6 Crude models for the associations of birth weight, gestational age and infant weight gain with preschool wheezing and school-age asthma

Table E7 Intermediate models of associations of birth weight, gestational age and infant weight gain with preschool wheezing and school-age asthma

Table E8 Associations of gestational age, birth weight, and infant weight gain with preschool wheezing in all countries (as presented in Figure 2 in the main manuscript), and in North-West European cohorts only

Table E9 Associations of gestational age, birth weight, and infant weight gain with school-age asthma in all countries (as presented in Figure 2 in the main manuscript 2), and in North-West European cohorts only

Table E10 Sensitivity analyses of associations of gestational age at birth and birth weight with preschool wheezing

Table E11 Sensitivity analyses of associations of gestational age at birth and birth weight with asthma

Figure E1 Flowchart of participating cohorts

Figure E2 Associations of birth weight with preschool wheezing and school-age asthma

**Table E1. Data collection on early growth characteristics and respiratory outcomes per cohort**

| **Cohort name**  **(country)** | **Birth weight** | **Gestational age** | **Weight at 1 year** | **Preschool wheezing** | **School-age asthma** | **Covariates and intermediates** |
| --- | --- | --- | --- | --- | --- | --- |
| ABIS  (Sweden) | Parental report | Parental report | Parental report | Parental report  ISAAC based questionnaire at ages 1, 2-3 years | Parental report  ISAAC based questionnaire at ages 5-6, 8-9 years | Parental questionnaires |
| ALSPAC  (United Kingdom) | Measured by research midwife | Medical record abstraction | Maternal report from community child health record | Annual questionnaires to mother from 6 months to 42 months | Maternal reported doctor-diagnosed asthma at 6½ years | Questionnaires at 18 weeks and 32 weeks gestation and annually from 6 months of age∙ |
| BILD  (Switzerland) | Midwife or gynaecologist record | Midwife or gynaecologist record | Measured at study visit  (age 5 weeks) | Standardized weekly telephone interview during first year of life asking for respiratory symptoms (runny nose, cough, wheeze, other) | (data not included in this meta-analysis) | Standardized questionnaire, midwife or gynaecologist record, standardized weekly telephone interviews |
| CONER  (Italy) | Interviews to the mothers | Interviews to the mothers | Collected by phone interviews to the mothers, based on the last measure taken during the last visit in the health care system | Collected by phone interviews to the mothers | Not collected | Questionnaires at birth, and phone interviews at 6m, 15m and 36m |
| COPSAC  (Denmark) | Midwife or gynaecologist record | Midwife or gynaecologist record | Measured at research unit | Propectively diary cards and diagnosed by research doctor | Diagnosed at research unit according to predefined algorithms | Interview to predefined questions and response categories |
| CZECH  (Czech) | Pediatrician | Pediatrician | NA | NA | Pediatrician  + allergologist | Pediatrician + maternal questionnaires |
| DNBC  (Denmark) | The Danish Medical Birth Register | The Danish National Patient Register | Computer-assisted telephone interview, age 18 months | ISAAC based computer-assisted telephone interview, age 18 months  Has he/she had episodes with wheezing respiration? | ISAAC based questionnaire, physician diagnosed asthma ever, age 7 years  Has a doctor ever said that your daughter had asthma? | Computer-assisted telephone interview week 12-16 of gestation, age 6 and 18 months |
| EDEN  (France) | Midwife | Obstetric record | Clinical exam performed by a midwife | ISAAC based questionnaire  at 4, 8, 12 months, 2 years, 3 years, 4years, 5 years | ISAAC based questionnaire 5 | Questionnaires and clinical exams during pregnancy and at 1 year |
| GASPII  (Italy) | Medical records | Medical records | Measured by pediatrician | ISAAC based questionnaire questionnaires  Age 15 months, 4 years | NA | Questionnaires at birth, 6 months, 15 months, 4 years |
|  |  |  |  |  |  |  |
| **Cohort name**  **(country)** | **Birth weight** | **Gestational age** | **Weight at 1 year** | **Preschool wheezing** | **School-age asthma** | **Covariates and intermediates** |
| GECKO Drenthe  (The Netherlands) | Parent-reported and  Midwife or gynaecologist record | Parent-reported and  Midwife or gynaecologist record | Measured by trained staff at Well Baby Clinic | Questionnaire at 14 months: Has your child suffered from wheezing breathing in the previous 3 months?  Questionnaire, at 45 months: Has your child ever suffered from wheezing in the chest?  Has your child suffered from wheezing in the chest in the previous 12 months?  How many attacks of wheezing in the chest has your child had during the previous 12 months? | Questionnaire at 45 months:  Was your child ever diagnosed with asthma by a doctor?  How old was your child when first diagnosed with asthma?  Did your child have asthma in the previous 12 months? | Midwife or gynaecologist record∙  Questionnaires  3^rd^ trimester of pregnancy, and at ages 2 weeks and 1/2/3/4/6/7/9/11/14 months |
| GENERATION R  (The Netherlands) | Midwife or gynaecologist record | Midwife or gynaecologist record | Measured at community health centre | ISAAC based questionnaire,  age 1 year  Has you child ever suffered from a whistling noise in the chest? | ISAAC based questionnaire, physician diagnosed asthma ever, age 6 years  Was your child ever diagnosed with asthma by a doctor? | Questionnaires  1^st^-3^rd^ trimester of pregnancy, age 1 year, age 2 years |
| GENERATION XXI  (Portugal) | Medical records | Medical records | From children health booklets: measured at community health centre | ISAAC based questionnaire, age 4-5 years  Has you child ever suffered from a whistling noise in the chest? | ISAAC based questionnaire, physician diagnosed asthma ever, age 4-5 years  Was your child ever diagnosed with asthma by a doctor? | Face-to-face structured questionnaires: birth, age 15 months, age 4-5 years |
| HUMIS  (Norway) | Medical Birth Registry | Medical Birth Registry  (136 newborns were intentionally oversampled, which results in a higher rate of preterm in the cohort than in the Norwegian population) | Maternal reports on measurement from community health centers | Has your child had any of the following diseases bronchitis? RS virus? (*In Norway there is no word for wheeze)∙  Doctor-diagnosed? (yes/no) | NA | MBR & questionnaire 1 month after delivery |
| INMA Gipuzkoa  (Spain) | Midwife | Self reported and confirmed by ultrasound from hospital records | Measured using a mechanical personal scale | ISAAC based questionnaire,  age 1 year  Has you child ever suffered from a whistling noise in the chest? | NA | Questionnaires  1^st^-3^rd^ trimester of pregnancy, age 1 year |
| INMA Menorca  (Spain) | Midwife or gynaecologist record | Midwife or gynaecologist record | Obtained from medical records | ISAAC based questionnaire,  age 1 year  Has you child ever suffered from a whistling noise in the chest? | ISAAC based questionnaire, physician diagnosed asthma ever, age 6 years  Was your child ever diagnosed with asthma by a doctor? | Questionnaires  of pregnancy, yearly 1 to 4 years, age 6 years |
| INMA Sabadell  (Spain) | Midwife | Self reported and confirmed by ultrasound from hospital records | Measured using a mechanical personal scale | ISAAC based questionnaire,  age 1 year  Has you child ever suffered from a whistling noise in the chest? | NA | Questionnaires  1^st^-3^rd^ trimester of pregnancy, age 1 year, age 2 years,  age 3-4 years |
| **Cohort name**  **(country)** | **Birth weight** | **Gestational age** | **Weight at 1 year** | **Preschool wheezing** | **School-age asthma** | **Covariates and intermediates** |
| INMA Valencia  (Spain) | Midwife | Self reported and confirmed by ultrasound from hospital records | Measured using a mechanical personal scale | ISAAC based questionnaire,  age 1 year  Has you child ever suffered from a whistling noise in the chest? | NA | Questionnaires  1^st^-3^rd^ trimester of pregnancy, age 1 year, age 2 years |
| ISLE OF WIGHT  (United Kingdom) | Midwife or gynaecologist record | Midwife or gynaecologist record | Measured at research clinic | Questionnaire  Has your child had wheeze in the last 12 months? | ISAAC based questionnaire, physician diagnosed asthma ever, age 10 and 18 years | Questionnaires  at birth, age 1 year, age 2 years, age 4 years, age 10 years and age 18 years |
| KOALA  (The Netherlands) | Midwife records and parental questionnaire | Midwife records and parental questionnaire | Parental questionnaire | ISAAC based questionnaire,  age 7 and 12 months  Has you child ever (or since last follow-up) suffered from a whistling noise in the chest? | ISAAC based questionnaire, physician diagnosed asthma ever, age 6-7 years  Was your child ever diagnosed with asthma by a doctor? | Questionnaires  34 weeks of pregnancy, age 7 months, age 1, 2, 4-5, 6-7 years |
| LEICESTER 1990  (United Kingdom) | Leicestershire  Health Authority Child Health Database: Birth notification and perinatal details | Leicestershire  Health Authority Child Health Database: Birth notification and perinatal details | NA | Has your child ever had attacks of wheezing? | Has any doctor or hospital told you that he/she has asthma or bronchitis? | Questionnaires 1990 (1-5 yrs old) and in 1998 (8-13 yrs) |
| LEICESTER 1998  (United Kingdom) | Leicestershire  Health Authority Child Health Database: Birth notification and perinatal details | Leicestershire  Health Authority Child Health Database: Birth notification and perinatal details | Leicestershire  Health Authority Child Health Database:  Health visitor records | Cohort 1998a:  Has your child ever had attacks of wheezing? Cohort 1998b:  ISAAC based questionnaire,  age 1 year Has your child ever suffered from a whistling noise in the chest? | Have you ever been told by a doctor or nurse that your child had asthma? | Questionnaires in 1998 (1-4 yrs) and in 2003 (6-10 yrs) |
| LIFEWAYS  (Ireland) | Maternal and neonatal hospital records | Maternal and neonatal hospital records | NA | NA | ISAAC adapted question – asthma diagnosed at age 5 years and or age 10 years  ‘Has a diagnosis of asthma ever been made in your Lifeways child?’ | Baseline questionnaire at ante-natal stage, mother and baby hospital records, questionnaires year 5 and year 10 follow-up |
| MAS  (Germany) | Infant’s “yellow booklet” | Gynaecologist record in yellow booklet | Follow-up at centre | ISAAC based questionnaire | ISAAC based questionnaire | Interviews and questionnaires |
| NINFEA  (Italy) | Questionnaire completed by the mothers at 6 months of age of the child | Questionnaire completed by the mothers during pregnancy and at 6 months of age of the child | Questionnaire completed by the mothers at 18 months of age of the child | ISAAC based questionnaire:  Age 6 months “Did your child experience episodes of wheezing in the first 6 months of life?” | NA | Questionnaires  during pregnancy, age 6 months, age 18 months |
| NINFEA  (Italy)  (Continued) |  |  |  | Age 18 months  “Did your child experience episodes of wheezing between 6 and 18 months of life?” |  |  |
| **Cohort name**  **(country)** | **Birth weight** | **Gestational age** | **Weight at 1 year** | **Preschool wheezing** | **School-age asthma** | **Covariates and intermediates** |
| PCB  (Slovakia) | Birth record | Gynaecologist record | Weight at 6 and 16 months – measured by the regional paediatrician | Pediatric report, age 45 months Wheezing associated with bronchitis, or pneumonia within the last year | NA | Questionnaires at delivery, age 6 and 16 months |
| PIAMA  (The Netherlands) | Parental reported in questionnaire∙ | Parental reported in questionnaire∙ During pregnancy the mother reported the expected date of birth (which, in most cases, she must have obtained from a calculation made in the antenatal clinic based on last menstrual period)∙ At about 3 months after birth, parents reported the actual date of birth | Parental reported in questionnaire∙ In some cases copied from records obtained from well baby clinic (JGZ); otherwise measured by the parents themselves | Parental reported in questionnaire | Parental reported in questionnaire | Parental reported in questionnaire |
| REPRO PL  (Poland) | Midwife or gynaecologist record (questionnaire filled by midwife or gynaecologist | Midwife or gynaecologist record (questionnaire filled by midwife or gynaecologist | Measured at medical centre | ISAAC based questionnaire,  age 1 and at 2 years: Has you child ever suffered from a whistling noise in the chest? | NA | Questionnaires  1^st^, 2^nd^ and 3^rd^ trimester of pregnancy, age 1 year, age 2 years |
| RHEA  (Greece) | Midwife or gynaecologist record | Midwife or gynaecologist record | Parental answered questionnaire | ISAAC based questionnaire,  Has your child ever had wheezing or whistling in the chest since birth? | NA | Questionnaires  1^st^-3^rd^ trimester of pregnancy, age 9 months, |
| SEATON  (United Kingdom) | Database of birth records | Database of birth records | NA | ISAAC based questionnaire,  age 1 year  Has you child ever suffered from a whistling noise in the chest? | ISAAC based questionnaire, physician diagnosed asthma ever, age 5 years  Has your child ever been diagnosed with asthma by a doctor? | Questionnaires:  1^st^ trimester of pregnancy, age 6 months, 1, 2 and 5 years |
| SWS  (United Kingdom) | Measurement recorded at birth | Detailed algorithm based on LMP and where necessary fetal ultrasound data∙ | Measured by research nurse in the infant’s home | ISAAC-based questionnaire at 6, 12 and 36 months of life:  Has your child had any episodes of chestiness associated with wheezing or whistling in his/her chest? (includes wheezy bronchitis, asthma) | ISAAC questionnaire at 6 years  Has your children ever had asthma?  If yes,  Was asthma diagnosed by a doctor? | Questionnaires at 11 and 34 weeks gestation and at 6, 12 and 36 months of life |
| WHISTLER  (The Netherlands) | Reported by the parents (as reported in the midwife or gynaecologist record) | Reported by the parents (as reported in the midwife or gynaecologist record) | Reported by the parents (measured at community health centre) | Daily questionnaire during first year of life Did your child wheeze today (whistling sound from the chest, not from the upper airways/ throat)? ICPC codes reported by the GP | ISAAC based questionnaire, physician diagnosed asthma ever, age 5 years. Was your child ever diagnosed with asthma by a doctor? ICPC codes reported by the GP | Questionnaires  age 3-8 weeks |

**Table E2. Characteristics of cohorts: determinants**

| **Cohort name**  **(country)** | **N** | **Birth years** | **Preterm birth** | **Low birth weight** | **Birth weight (SDS)** |
| --- | --- | --- | --- | --- | --- |
|  | 147,252 |  | % (n) | % (n) | mean (SD) |
| ABIS  (Sweden) | 6,829 | 1997-1998 | 4∙1 (278) | 2∙8 (188) | 0∙31 (1∙0) |
| ALSPAC  (United Kingdom) | 12,485 | 1991-1992 | 5∙9 (738) | 5∙1 (626) | 0∙03 (1∙0) |
| BILD  (Switzerland) | 432 | 1999 | 1∙9 (8) | 1∙6 (7) | -0∙14 (1∙0) |
| CONER  (Italy) | 389 | 2004-2005 | 4∙9 (19) | 3∙1 (12) | -0∙09 (0∙9) |
| COPSAC  (Denmark) | 384 | 1998-2001 | 2∙9 (11) | 1∙8 (7) | 0∙12 (1∙0) |
| CZECH  (Czech) | 1,830 | 2001-2004 | 5∙7 (102) | 5∙4 (98) | -0∙13 (1∙0) |
| DNBC  (Denmark) | 76,810 | 1996-2001 | 4∙3 (3,338) | 2∙8 (2,033) | 0∙27 (1∙0) |
| EDEN  (France) | 1,774 | 2003-2005 | 5∙4 (95) | 5∙0 (89) | -0∙20 (0∙9) |
| GASPII  (Italy) | 694 | 2003-2004 | 6∙1 (42) | 5∙9 (41) | -0∙10 (1∙0) |
| GECKO Drenthe  (The Netherlands) | 1,718 | 2006-2007 | 4∙5 (74) | 2∙7 (45) | 0∙25 (1∙0) |
| GENERATION R  (The Netherlands) | 5,815 | 2002-2006 | 5∙7 (330) | 5∙3 (306) | -0∙04 (1∙0) |
| GENERATION XXI  (Portugal) | 7,053 | 2005-2006 | 9∙3 (656) | 9∙2 (651) | -0∙20 (0∙9) |
| HUMIS  (Norway) | 2,001 | 2003-2008 | 10∙5 (171) | 6∙8 (136) | 0∙28 (1∙1) |
| INMA Gipuzkoa  (Spain) | 478 | 2006-2008 | 3∙4 (16) | 4∙7 (22) | -0∙33 (0∙9) |
| INMA Menorca  (Spain) | 474 | 1997-1998 | 4∙9 (23) | 6∙5 (31) | -0∙44 (1∙0) |
| INMA Sabadell  (Spain) | 502 | 2004-2007 | 3∙0 (15) | 4∙2 (21) | -0∙44 (0∙9) |
| INMA Valencia  (Spain) | 604 | 2003-2005 | 4∙6 (28) | 5∙1 (31) | -0∙41 (1∙0) |
| ISLE OF WIGHT  (United Kingdom) | 1,405 | 1989-1990 | 2∙8 (40) | 3∙8 (53) | -0∙15 (1∙0) |
| KOALA  (The Netherlands) | 2,151 | 2000-2003 | 2∙9 (63) | 2∙4 (51) | 0∙11 (1∙0) |
| LEICESTER 1990  (United Kingdom) | 1,231 | 1990 | 5∙6 (24) | 5∙7 (70) | -0∙00 (1∙0) |
| LEICESTER 1998  (United Kingdom) | 6,836 | 1998 | 6∙4 (437) | 7∙3 (497) | -0∙14 (1∙1) |
| LIFEWAYS  (Ireland) | 421 | 2001-2002 | 4∙4 (17) | 4∙3 (18) | 0∙16 (1∙1) |
| MAS  (Germany) | 1,263 | 1990 | 3∙1 (38) | 2∙7 (34) | -0∙17 (0∙9) |
| NINFEA  (Italy) | 1,922 | 2005-2010 | 7∙3 (140) | 6∙4 (120) | -0∙38 (1∙0) |
| PCB  (Slovakia) | 429 | 2001-2004 | 1∙2 (5) | 3∙5 (15) | -0∙26 (1∙0) |
| PIAMA  (The Netherlands) | 3,631 | 1996-1997 | 4∙8 (173) | 3∙4 (122) | 0∙13 (1∙0) |
| REPRO PL  (Poland) | 314 | 2007-2011 | 5∙1 (16) | 3∙8 (11) | -0∙03 (1∙0) |
| RHEA  (Greece) | 1,046 | 2007-2008 | 11∙9 (124) | 5∙1 (53) | -0∙01 (0∙9) |
| SEATON  (United Kingdom) | 1,891 | 1997 | 7∙8 (148) | 5∙3 (97) | 0∙10 (1∙0) |
| SWS  (United Kingdom) | 2,291 | 1998-2007 | 6∙3 (145) | 4∙3 (98) | 0∙00 (1∙0) |
| WHISTLER  (The Netherlands) | 2,149 | 2001-2012 | 3∙3 (70) | 2∙7 (57) | 0∙16 (1∙0) |

Preterm birth was defined as a gestational age < 37 weeks, low birth weight was defined as a birth weight < 2500 grams.

**Table E3. Characteristics of cohorts: confounders**

| **Cohort name (country)** | **Educational level** | | | | **Prenatal smoke** | | **Maternal asthma** | | **Postnatal smoke** | | **Sex** | | **Siblings** | | **Day care** | |
| --- | --- | --- | --- | --- | --- | --- | --- | --- | --- | --- | --- | --- | --- | --- | --- | --- |
|  | Low | Medium | High | Missing | Yes | Missing | Yes | Missing | Yes | Missing | Female | Missing | Yes | Missing | Yes | Missing |
| ABIS  (Sweden) | 6∙3  (430) | 58∙5  (3,983) | 35∙2  (2,396) | 0∙3  (20) | 8∙0  (545) | 0∙4  (24) | - | - | 10∙6  (715) | 0∙8  (56) | 48∙4  (3,303) | 0  (0) | 60∙2  (4,098) | 0∙4  (24) | 3∙6  (223) | 9∙2  (629) |
| ALSPAC  (United Kingdom) | 63∙7  (7,488) | 23∙0  (2,701) | 13∙3  (1,566) | 5∙8  (730) | 23∙7  (2,862) | 3∙4  (425) | 11∙4  (1,343) | 5∙8  (723) | 23∙9  (2,657) | 11∙0  (1,376) | 48∙3  (6,031) | 0  (0) | 55∙1  (6,526) | 5∙1  (631) | 3∙0  (374) | 12∙3  (1,535) |
| BILD  (Switzerland) | 33∙1  (139) | 37∙9  (159) | 29∙0  (122) | 2∙8  (12) | 9∙5  (41) | 0  (0) | 10∙2  (44) | 0∙5  (2) | - | - | 54∙2  (234) | 0  (0) | 53∙5  (230) | 0∙5  (2) | - | - |
| CONER  (Italy) | 15∙5  (60) | 46∙1  (179) | 38∙4  (149) | 0∙3  (1) | 11∙3  (43) | 2∙1  (8) | 7∙5  (29) | 0  (0) | 6∙9  (27) | 0  (0) | 49∙4  (192) | 0  (0) | 43∙4  (169) | 0  (0) | - | - |
| COPSAC  (Denmark) | 59∙8  (220) | 26∙1  (96) | 14∙1  (52) | 4∙2  (16) | 14∙6  (56) | 0  (0) | 100  (384) | 0  (0) | 18∙9  (66) | 8∙9  (34) | 50∙0  (192) | 0  (0) | 40∙6  (152) | 2∙6  (10) | 56∙3  (206) | 4∙7  (18) |
| CZECH  (Czech) | 13∙6  (249) | 35∙7  (652) | 50∙7  (925) | 0∙2  (4) | 16∙5  (302) | 0∙1  (2) | 3∙3  (60) | 0∙1  (1) | 21∙6  (395) | 0∙1  (2) | 49∙1  (899) | 0  (0) | 50∙2  (918) | 0∙1  (2) | - | - |
| DNBC  (Denmark) | 8∙8  (6,466) | 37∙6  (27,580) | 53∙6  (39,317) | 4∙5  (3,447) | 24∙0  (18,414) | 4∙2  (3,227) | 8∙4  (6,186) | 4∙3  (3,295) | 18∙0  (11,151) | 19∙4  (14,925) | 48∙9  (37,566) | 0  (0) | 53∙5  (39,387) | 4∙2  (3,233) | 89∙0  (58,492) | 14∙5  (11,120) |
| EDEN  (France) | 6∙1  (107) | 61∙1  (1,067) | 32∙7  (571) | 1∙6  (29) | 25∙9  (456) | 0∙9  (16) | 10∙9  (192) | 0∙3  (5) | 19∙8  (351) | 0  (0) | 48∙0  (851) | 0  (0) | 68∙7  (43) | 22∙7  (402) | 12∙0  (212) | 0 (0) |
| GASPII  (Italy) | 13∙6  (94) | 50∙4  (348) | 35∙9  (248) | 0∙6  (4) | 12∙5  (86) | 0∙6  (4) | 11∙0  (76) | 0  (0) | 3∙6  (25) | 0  (0) | 49∙7  (345) | 0  (0) | 41∙3  (286) | 0∙1  (1) | 4∙5  (31) | 0 (0) |
| GECKO Drenthe  (The Netherlands) | 0∙7  (8) | 62∙7  (698) | 36∙6  (408) | 35∙2  (604) | 14∙3  (238) | 3∙4  (58) | - | - | 14∙6  (202) | 19∙7  (339) | 49∙4  (843) | 0∙6  (11) | 69∙2  (1,072) | 9∙8  (169) | 32∙6  (515) | 8∙0  (137) |
| GENERATION R  (The Netherlands) | 6∙4  (352) | 38∙7  (2,135) | 54∙9  (3,027) | 5∙2  (301) | 13∙7  (715) | 10∙1  (585) | 7∙7  (393) | 11∙7  (683) | 14∙6  (268) | 68∙4  (3,978) | 50∙3  (2,924) | 0  (1) | 42∙0  (2,375) | 2∙9  (166) | 58∙0  (2,677) | 20∙6  (1,197) |
| GENERATION XXI  (Portugal) | 22∙8  (1,594) | 51∙7  (3,614) | 25∙5  (1,786) | 0∙8  (59) | 13∙5  (900) | 5∙2  (365) | 5∙3  (359) | 4∙4  (307) | - | - | 49∙1  (3,464) | 0  (0) | 42∙0  (2,906) | 1∙8  (129) | 21∙6  (194) | 87∙3  (6,156) |
| HUMIS  (Norway) | 14∙2  (279) | 23∙2  (455) | 62∙6  (1,226) | 2∙0  (41) | 12∙2  (243) | 0∙7  (15) | 7∙0  (138) | 1∙0  (20) | 8∙7  (116) | 33∙3  (666) | 48∙0  (892) | 0  (0) | 60∙5  (982) | 18∙9  (379) | 18∙3  (342) | 6∙8  (137) |
| INMA Gipuzkoa  (Spain) | 12∙6  (60) | 37∙4  (179) | 50∙0  (239) | 0  (0) | 24∙9  (116) | 2∙5  (12) | 6∙5  (31) | 0  (0) | 20∙0  (95) | 0∙4  (2) | 51∙4  (245) | 0  (0) | 44∙1  (211) | 0  (0) | 47∙1  (224) | 0∙4  (2) |
| INMA Menorca  (Spain) | 58∙5  (268) | 28∙2  (129) | 13∙3  (61) | 3∙4  (16) | 37∙6  (178) | 0  (0) | 5∙9  (28) | 0∙2  (1) | 30∙0  (142) | 0  (0) | 48∙5  (230) | 0  (0) | 50∙8  (241) | 0  (0) | 23∙5  (110) | 1∙1  (5) |
| INMA Sabadell  (Spain) | 25∙7  (128) | 43∙1  (215) | 31∙3  (156) | 0∙6  (3) | 30∙2  (150) | 1∙2  (6) | 8∙2  (41) | 0∙2  (1) | 27∙6  (137) | 1∙0  (5) | 47∙0  (236) | 0  (0) | 43∙8  (219) | 0∙4  (2) | 32∙3  (160) | 1∙2  (6) |
| **Cohort name (country)** | **Educational level** | | | | **Prenatal smoke** | | **Maternal asthma** | | **Postnatal smoke** | | **Sex** | | **Siblings** | | **Day care** | |
|  | Low | Medium | High | Missing | Yes | Missing | Yes | Missing | Yes | Missing | Female | Missing | Yes | Missing | Yes | Missing |
| INMA Valencia  (Spain) | 30∙1  (182) | 44∙4  (268) | 25∙5  (154) | 0  (0) | 40∙7  (246) | 0  (0) | 8∙0  (48) | 0∙2  (1) | 31∙8  (189) | 1∙5  (9) | 47∙4  (286) | 0  (0) | 45∙2  (273) | 0  (0) | 22∙1  (132) | 1∙3  (8) |
| ISLE OF WIGHT  (United Kingdom) | 0  (0) | 0  (0) | - | - | 23∙3  (325) | 0∙7  (10) | 10∙2  (143) | 0∙4  (6) | - | - | 49∙4  (694) | 0  (0) | 51∙1  (597) | 16∙9  (237) | - | - |
| KOALA  (The Netherlands) | 3∙8  (81) | 45∙5  (967) | 50∙7  (1,077) | 1∙2  (26) | 6∙1  (131) | 0∙3  (6) | 9∙4  (201) | 0∙9  (20) | - | - | 49∙3  (1,061) | 0  (0) | 55∙4  (1,181) | 0∙9  (19) | 60∙3  (1,289) | 0∙6  (13) |
| LEICESTER 1990  (United Kingdom) | 0  (0) | 0  (0) | - | - | 30∙3  (363) | 2∙6  (32) | - | - | - | - | 49∙4  (608) | 0  (0) | 63∙6  (272) | 65∙2  (803) | - | - |
| LEICESTER 1998  (United Kingdom) | 42∙0  (1,198) | 35∙6  (1,016) | 22∙4  (640) | 58∙3  (3,982) | 16∙1  (1,000) | 8∙9  (607) | 19∙9  (1,218) | 10∙6  (728) | - | - | 48∙1  (3,289) | 0  (0) | 58∙6  (3,882) | 3∙0  (206) | - | - |
| LIFEWAYS  (Ireland) | 0  (0) | 1∙1  (2) | 98∙9  (174) | 58∙2  (245) | 18∙3  (77) | 0∙2  (1) | - | - | - | - | 53∙7  (226) | 0  (0) | 57∙7  (239) | 1∙7  (7) | 16∙4  (69) | 0  (0) |
| MAS  (Germany) | 9∙2  (112) | 53∙5  (650) | 37∙3  (454) | 3∙7  (47) | 25∙4  (320) | 0∙4  (5) | 8∙2  (102) | 1∙3  (16) | 28∙4  (351) | 2∙1  (27) | 47∙7  (603) | 0  (0) | 40∙8  (465) | 9∙8  (124) | 6∙6  (73) | 12∙4  (156) |
| NINFEA  (Italy) | 4∙1  (78) | 35∙6  (678) | 60∙3  (1,146) | 1∙0  (20) | 8∙7  (166) | 0∙5  (9) | 8∙0  (148) | 3∙9  (75) | 7∙8  (146) | 3∙1  (60) | 49∙9  (959) | 0  (0) | 22∙6  (434) | 0  (0) | 26∙2  (474) | 5∙8  (112) |
| PCB  (Slovakia) | 43∙6  (186) | 50∙8  (217) | 5∙6  (24) | 0∙5  (2) | - | - | 1∙8  (6) | 24∙0  (103) | - | - | 50∙3  (216) | 0  (0) | 59∙4  (255) | 0  (0) | - | - |
| PIAMA  (The Netherlands) | 22∙6  (814) | 42∙0  (1,512) | 35∙4  (1,275) | 0∙8  (30) | 16∙9  (610) | 0∙6  (23) | 7∙1  (256) | 0∙2  (7) | 13∙7  (492) | 0∙7  (27) | 48∙3  (1,752) | 0  (0) | 50∙3  (1,826) | 0∙1  (2) | 56∙3  (2,022) | 1∙1  (40) |
| REPRO PL  (Poland) | 5∙7  (18) | 30∙9  (97) | 63∙4  (199) | 0  (0) | 12∙1  (38) | 0  (0) | 1∙9  (6) | 0  (0) | 14∙1  (44) | 1∙0  (3) | 51∙9  (163) | 0  (0) | 41∙1  (129) | 0  (0) | 5∙9  (18) | 2∙5  (8) |
| RHEA  (Greece) | 18∙2  (182) | 51∙3  (513) | 30∙5  (305) | 4∙4  (46) | 21∙8  (218) | 4∙6  (48) | 3∙0  (29) | 8∙3  (87) | 28∙7  (298) | 0∙7  (7) | 49∙9  (522) | 0  (0) | 59∙3  (595) | 4∙1  (43) | 2∙4  (25) | 0∙2  (2) |
| SEATON  (United Kingdom) | 30∙4  (463) | 31∙0  (472) | 38∙6  (587) | 19∙5  (369) | 29∙6  (559) | 0∙1  (1) | 16∙7  (315) | 0∙1  (1) | 14∙6  (211) | 23∙5  (445) | 49∙8  (897) | 4∙7  (88) | 45∙7  (865) | 0  (0) | 44∙6  (417) | 50∙6  (957) |
| SWS  (United Kingdom) | 41∙6  (951) | 30∙1  (687) | 28∙3  (646) | 0∙3  (7) | 16∙9  (376) | 3∙0  (68) | 23∙9  (510) | 6∙9  (157) | 19∙5  (440) | 1∙3  (30) | 45∙8  (1,050) | 0  (0) | 50∙9  (1,164) | 0∙1  (2) | - | - |
| WHISTLER  (The Netherlands) | 7∙8  (134) | 26∙5  (452) | 65∙7  (1,122) | 20∙5  (441) | 6∙0  (130) | 0∙1  (3) | 8∙5  (144) | 21∙0  (452) | - | - | 50∙6  (1,087) | 0  (1) | 53∙1  (1,129) | 1∙0  (22) | 71∙0  (1,368) | 10∙3  (222) |

Values are valid percentages (absolute numbers) for the information of the confounders, and percentages (absolute numbers) for the amount of missing data

**Table E4. Characteristics of cohorts: intermediates**

| **Cohort name (country)** | **Ever breastfed** | | **Lower respiratory tract infections** | | **Eczema** | |
| --- | --- | --- | --- | --- | --- | --- |
|  | Yes | Missing | Yes | Missing | Yes | Missing |
| ABIS  (Sweden) | - | - | 6∙5  (379) | 14∙1  (962) | 22∙4  (1,270) | 17∙0  (1,164) |
| ALSPAC  (United Kingdom) | 73∙9  (8,053) | 12∙7  (1,585) | - | - | 45∙2  (5,024) | 10∙9  (1,365) |
| BILD  (Switzerland) | - | - | - | - | - | - |
| CONER  (Italy) | 90∙6  (349) | 1∙0  (4) | 29∙6  (115) | 0  (0) | 18∙3  (71) | 0  (0) |
| COPSAC  (Denmark) | 96∙9  (370) | 0∙5  (2) | 45∙2  (169) | 2∙6  (10) | 39∙6  (128) | 15∙9  (61) |
| CZECH  (Czech) | 90∙4  (1,652) | 0∙1  (2) | 38∙5  (704) | 0  (0) | 14∙5  (266) | 0  (0) |
| DNBC  (Denmark) | 99∙1  (53,336) | 29∙9  (22,995) | 20∙1  (13,157) | 14∙9  (11,430) | 9∙2  (5,495) | 22∙2  (17,016) |
| EDEN  (France) | 70∙9  (1,225) | 0∙2  (4) | 56∙0  (993) | 0  (0) | 38∙0  (657) | 0  (0) |
| GASPII  (Italy) | 88∙6  (615) | 0  (0) | 25∙6  (178) | 0  (0) | 21∙9  (152) | 0∙1  (1) |
| GECKO Drenthe  (The Netherlands) | 81∙5  (1,396) | 0∙3  (6) | - | - | 19∙1  (96) | 70∙8  (1,216) |
| GENERATION R  (The Netherlands) | 92∙1  (5,141) | 4∙0  (230) | 14∙0  (749) | 8∙1  (471) | 12∙9  (636) | 15∙0  (873) |
| GENERATION XXI  (Portugal) | 92∙9  (6,503) | 0∙7  (51) | 26∙6  (233) | 87∙6  (6,177) | 10∙7  (74) | 90∙2  (6,359) |
| HUMIS  (Norway) | 98∙5  (1,685) | 14∙5  (290) | 19∙5  (390) | 0  (0) | 26∙1  (522) | 0∙1  (2) |
| INMA Gipuzkoa  (Spain) | 84∙4  (342) | 15∙3  (73) | - | - | - | - |
| INMA Menorca  (Spain) | 82∙3  (390) | 0  (0) | 45∙9  (188) | 13∙5  (64) | 38∙3  (146) | 14∙1  (67) |
| INMA Sabadell  (Spain) | 86∙9  (370) | 15∙1  (76) | 63∙7  (312) | 2∙4  (12) | 36∙7  (178) | 3∙4  (17) |
| INMA Valencia  (Spain) | 70∙7  (353) | 17∙4  (105) | 47∙6  (280) | 2∙6  (16) | 30∙9  (186) | 0∙3  (2) |
| ISLE OF WIGHT  (United Kingdom) | 77∙6  (972) | 10∙9  (153) | 8∙0  (95) | 15∙7  (220) | 24∙9  (295) | 15∙8  (222) |
| KOALA  (The Netherlands) | 85∙5  (1,840) | 0  (0) | - | - | 32∙0  (681) | 0  (0) |
| LEICESTER 1990  (United Kingdom) | 55∙9  (264) | 61∙7  (759) | - | - | - | - |
| LEICESTER 1998  (United Kingdom) | 60∙0  (3,829) | 6∙7  (455) | 19∙0  (754) | 41∙8  (2,860) | - | - |
| LIFEWAYS  (Ireland) | 61∙8  (260) | 0  (0) | - | - | - | - |
| MAS  (Germany) | 91∙6  (1,155) | 0∙2  (2) | 38∙1  (368) | 23∙6  (298) | - | - |
| NINFEA  (Italy) | 90∙4  (1,689) | 2∙8  (53) | 24∙7  (439) | 7∙4  (143) | 24∙3  (493) | 6∙0  (115) |
| PCB  (Slovakia) | 2∙1  (9) | 0∙9  (4) | - | - | - | - |
| PIAMA  (The Netherlands) | 82∙5  (2,962) | 1∙1  (39) | 22∙9  (793) | 4∙7  (169) | 23∙5  (832) | 2∙7  (97) |
| REPRO PL  (Poland) | 93∙9  (295) | 0  (0) | 40∙8  (128) | 0  (0) | 20∙2  (63) | 0∙6  (2) |
| **Cohort name (country)** | **Ever breastfed** | | **Lower respiratory tract infections** | | **Eczema** | |
|  | Yes | Missing | Yes | Missing | Yes | Missing |
| RHEA  (Greece) | 85∙2  (890) | 0∙1  (1) | 21∙5  (225) | 0  (0) | 13∙6  (140) | 2∙5  (58) |
| SEATON  (United Kingdom) | 69∙9  (1,117) | 15∙4  (292) | - | - | 26∙3  (441) | 11∙4  (216) |
| SWS  (United Kingdom) | 81∙5  (1,809) | 3∙1  (72) | 25∙4  (569) | 2∙3  (53) | 13∙6  (304) | 2∙5  (58) |
| WHISTLER  (The Netherlands) | 77∙5  (1,660) | 0∙4  (8) | 35∙7  (755) | 1∙7  (36) | 24∙3  (513) | 1∙7  (36) |

Values are valid percentages (absolute numbers) for the information of the intermediates, and percentages (absolute numbers) for the amount of missing data

**Table E5. Confounder models of associations of birth weight, gestational age and infant weight gain with preschool wheezing and school-age asthma**

|  | **Pooled odds ratios**  **random effects** | **Q-value** | **p-value** | **I^2^** |
| --- | --- | --- | --- | --- |
|  | **Preschool wheezing** | | | |
| Gestational age at birth (week) | 0∙95 (0∙94, 0∙95)*** | 27∙06 | 0∙515 | 0∙00 |
| Gestational age at birth, adjusted for birth weight (week) | 0∙95 (0∙94, 0∙95)*** | 24∙79 | 0∙640 | 0∙00 |
| Preterm birth (<37 weeks vs. >=37 weeks) | 1∙34 (1∙25, 1∙43)*** | 27∙75 | 0∙424 | 2∙70 |
| Birth weight (500 gram) | 0∙95 (0∙93, 0∙96)*** | 32∙57 | 0∙252 | 14∙02 |
| Birth weight, adjusted for gestational age at birth (500 gram) | 1∙00 (0∙98, 1∙02) | 30∙84 | 0∙324 | 9∙20 |
| Low birth weight (<2500 grams vs.>= 2500 grams) | 1∙37 (1∙27, 1∙27)*** | 27∙27 | 0∙343 | 8∙32 |
| Low birth weight, adjusted for gestational age at birth (<2500 grams vs. >= 2500 grams) | 1∙10 (1∙00, 1∙21)* | 29∙48 | 0∙244 | 15∙20 |
| Infant weight gain (100 gram per month) | 1∙13 (1∙10, 1∙15)*** | 37∙03 | 0∙057 | 32∙48 |
|  | **School-age asthma** | | | |
| Gestational age at birth (week) | 0∙94 (0∙91, 0∙95)*** | 32∙95 | 0∙017 | 45∙37 |
| Gestational age at birth, adjusted for birth weight (week) | 0∙94 (0∙91, 0∙97)*** | 45∙36 | <0∙001 | 60∙31 |
| Preterm birth (<37 weeks vs. >=37 weeks) | 1∙40 (1∙18, 1∙67)*** | 29∙07 | 0∙034 | 41∙52 |
| Birth weight (500 gram) | 0∙92 (0∙90, 0∙95)*** | 26∙30 | 0∙093 | 31∙55 |
| Birth weight, adjusted for gestational age at birth (500 gram) | 0∙99 (0∙94, 1∙04) | 38∙28 | 0∙004 | 52∙97 |
| Low birth weight (<2500 grams vs. >= 2500 grams) | 1∙54 (1∙40, 1∙69)*** | 11∙47 | 0∙718 | 0∙00 |
| Low birth weight, adjusted for gestational age at birth (<2500 grams vs. >= 2500 grams) | 1∙13 (1∙01, 1∙27)* | 10∙50 | 0∙787 | 0∙00 |
| Infant weight gain (100 gram per month) | 1∙10 (1∙04 to1∙16)*** | 39∙52 | 0∙001 | 62∙04 |

Values are pooled odds ratios (95% confidence interval) from random effect models. Models are adjusted for maternal educational level, smoking during pregnancy, history of asthma, and smoking during infancy, and for child’s sex, siblings, and attending day care. Infant weight gain is additionally adjusted for gestational age at birth and birth weight. *p<0∙05, **p<0∙01, ***p<0∙001

**Table E6.** **Crude models of associations of birth weight, gestational age and infant weight gain with preschool wheezing and school-age asthma**

|  | **Pooled odds ratios**  **random effects** | **Q-value** | **p-value** | **I^2^** |
| --- | --- | --- | --- | --- |
|  | **Preschool wheezing** | | | |
| Gestational age at birth (week) | 0∙95 (0∙94, 0∙96)** | 30∙30 | 0∙349 | 7∙58 |
| Gestational age at birth, adjusted for birth weight (week) | 0∙95 (0∙94, 0∙96)** | 31∙57 | 0∙293 | 11∙29 |
| Preterm birth (<37 weeks vs. >=37 weeks) | 1∙30 (1∙23, 1∙38)** | 24∙55 | 0∙600 | 0∙00 |
| Birth weight (500 gram) | 0∙96 (0∙94, 0∙97)** | 40∙70 | 0∙057 | 31∙20 |
| Birth weight, adjusted for gestational age at birth (500 gram) | 1∙01 (0∙98, 1∙03) | 43∙25 | 0∙032 | 35∙40 |
| Low birth weight (<2500 grams vs. >= 2500 grams) | 1∙34 (1∙24, 1∙45)** | 29∙82 | 0∙231 | 16∙17 |
| Low birth weight, adjusted for gestational age at birth (<2500 grams vs. >= 2500 grams) | 1∙08 (0∙98, 1∙19) | 30∙40 | 0∙210 | 17∙77 |
| Infant weight gain (100 gram per month) | 1∙12 (1∙09, 1∙15)*** | 38∙92 | 0∙038 | 35∙77 |
|  | **School-age asthma** | | | |
| Gestational age at birth (week) | 0∙93 (0∙91, 0∙95)** | 41∙01 | 0∙002 | 56∙11 |
| Gestational age at birth, adjusted for birth weight (week) | 0∙94 (0∙91, 0∙97)** | 46∙96 | 0∙000 | 61∙68 |
| Preterm birth (<37 weeks vs. >=37 weeks) | 1∙41 (1∙18, 1∙68)** | 29∙94 | 0∙027 | 43∙21 |
| Birth weight (500 gram) | 0∙92 (0∙89, 0∙95)** | 36∙86 | 0∙005 | 51∙17 |
| Birth weight, adjusted for gestational age at birth (500 gram) | 0∙98 (0∙93, 1∙03) | 41∙54 | 0∙001 | 56∙67 |
| Low birth weight (<2500 grams vs. >= 2500 grams) | 1∙55 (1∙38, 1∙75)** | 17∙66 | 0∙281 | 15∙07 |
| Low birth weight, adjusted for gestational age at birth (<2500 grams vs. >= 2500 grams) | 1∙18 (1∙05, 1∙32)** | 9∙89 | 0∙827 | 0∙00 |
| Infant weight gain (100 gram per month) | 1∙11 (1∙05, 1∙17)*** | 38∙29 | 0∙001 | 60∙83 |

Values are pooled odds ratios (95% confidence interval) from random effect models. Models are adjusted for child’s sex∙ Infant weight gain is additionally adjusted for gestational age at birth and birth weight. *p<0∙05, **p<0∙01, ***p<0∙001

**Table E7.**  **Intermediates models of associations of birth weight, gestational age and infant weight gain with preschool wheezing and school-age asthma**

|  | **Pooled odds ratios**  **random effects model** | **Q-value** | **p-value** | **I^2^** |
| --- | --- | --- | --- | --- |
|  | **Preschool wheezing** | | | |
| Gestational age at birth (week) | 0∙95 (0∙95, 0∙96)** | 22∙82 | 0∙695 | 0∙00 |
| Gestational age at birth, adjusted for birth weight (week) | 0∙95 (0∙95, 0∙96)** | 22∙17 | 0∙729 | 0∙00 |
| Preterm birth (<37 weeks vs. >=37 weeks) | 1∙27 (1∙19, 1∙36)** | 26∙22 | 0∙451 | 0∙84 |
| Birth weight (500 gram) | 0∙96 (0∙94, 0∙97)** | 27∙77 | 0∙423 | 2∙77 |
| Birth weight, adjusted for gestational age at birth (500 gram) | 1∙00 (0∙98, 1∙02) | 28∙14 | 0∙404 | 4∙04 |
| Low birth weight (<2500 grams vs. >= 2500 grams) | 1∙32 (1∙23, 1∙43)** | 25∙20 | 0∙395 | 4∙75 |
| Low birth weight, adjusted for gestational age at birth (<2500 grams vs. >= 2500 grams) | 1∙09 (0∙99, 1∙19) | 27∙13 | 0∙298 | 11∙54 |
| Infant weight gain (100 gram per month) | 1∙12 (1∙09, 1∙15)*** | 33∙44 | 0∙120 | 25∙24 |
|  | **School-age asthma** | | | |
| Gestational age at birth (week) | 0∙94 (0∙92, 0∙96)** | 33∙41 | 0∙015 | 46∙13 |
| Gestational age at birth, adjusted for birth weight (week) | 0∙94 (0∙91, 0∙97)** | 41∙41 | 0∙001 | 56∙53 |
| Preterm birth (<37 weeks vs. >=37 weeks) | 1∙36 (1∙14, 1∙63)** | 29∙01 | 0∙034 | 41∙39 |
| Birth weight (500 gram) | 0∙93 (0∙90, 0∙96)** | 29∙24 | 0∙046 | 38∙43 |
| Birth weight, adjusted for gestational age at birth (500 gram) | 0∙99 (0∙94, 1∙04) | 37∙74 | 0∙004 | 52∙30 |
| Low birth weight (<2500 grams vs. >= 2500 grams) | 1∙50 (1∙36, 1∙65)** | 11∙32 | 0∙730 | 0∙00 |
| Low birth weight, adjusted for gestational age at birth (<2500 grams vs. >= 2500 grams) | 1∙12 (1∙00, 1∙26) | 9∙80 | 0∙832 | 0∙00 |
| Infant weight gain (100 gram per month) | 1∙09 (1∙03, 1∙15)** | 36∙15 | 0∙002 | 58∙50 |

Values are pooled odds ratios (95% confidence interval) from random effect models. Models are adjusted for maternal educational level, smoking during pregnancy, history of asthma, and smoking during infancy, and for child’s sex, siblings, attending day care, breastfeeding status, lower respiratory tract infections, and eczema. Infant weight gain is additionally adjusted for gestational age at birth and birth weight. *p<0∙05, **p<0∙01, ***p<0∙001

**Table E8. Associations of gestational age, birth weight, and infant weight gain with preschool wheezing in all countries (as presented in Figure 2 in the main manuscript), and in North-West European cohorts only**

|  |  |  | **TOTAL EUROPE** |  |  |  | **NORTH-WEST EUROPE** |  |
| --- | --- | --- | --- | --- | --- | --- | --- | --- |
|  |  |  | **(n = 129,813)** |  |  |  | **(n = 117,352)** |  |
|  |  | **n** | **Odds Ratio (95% CI)** | **p-value** |  | **n** | **Odds Ratio (95% CI)** | **p-value** |
| **Gestational age** | **Birth weight** |  |  |  |  |  |  |  |
| **<32 weeks** | **<-2 SD** | 19 | 2∙59 (1∙02, 6∙53) | 0∙044 |  | 18 | 2∙99 (1∙16, 7∙70) | 0∙024 |
|  | **-2 to -1 SD** | 71 | 0∙98 (0∙58, 1∙65) | 0∙932 |  | 59 | 1∙17 (0∙67, 2∙06) | 0∙582 |
|  | **-1 to 1 SD** | 325 | 2∙18 (1∙73, 2∙74) | <0∙001 |  | 282 | 2∙47 (1∙93, 3∙15) | <0∙001 |
|  | **1 to 2 SD** | 42 | 2∙16 (1∙15, 4∙07) | 0∙017 |  | 39 | 1∙97 (1∙02, 3∙78) | 0∙043 |
|  | **>=2 SD** | 10 | 1∙31 (0∙32, 5∙35) | 0∙711 |  | 8 | 1∙74 (0∙39, 7∙84) | 0∙470 |
| **32-36 weeks** | **<-2 SD** | 93 | 1∙32 (0∙85, 2∙05) | 0∙213 |  | 80 | 1∙20 (0∙74, 1∙94) | 0∙458 |
|  | **-2 to -1 SD** | 360 | 1∙47 (1∙18, 1,83) | 0∙001 |  | 300 | 1∙47 (1∙15, 1∙87) | 0∙002 |
|  | **-1 to 1 SD** | 1992 | 1∙54 (1∙40, 1∙69) | <0∙001 |  | 1702 | 1∙56 (1∙40, 1∙73) | <0∙001 |
|  | **1 to 2 SD** | 365 | 1∙72 (1∙39, 2∙14) | <0∙001 |  | 317 | 1∙75 (1∙93, 2∙20) | <0∙001 |
|  | **>=2 SD** | 110 | 1∙35 (0∙90, 2∙01) | 0∙143 |  | 85 | 1∙54 (0∙98, 2∙40) | 0∙058 |
| **36-40 weeks** | **<-2 SD** | 1145 | 1∙12 (0∙98, 1∙27) | 0∙095 |  | 955 | 1∙14 (0∙99, 1∙31) | 0∙077 |
|  | **-2 to -1 SD** | 5458 | 1∙17 (1∙10, 1∙25) | <0∙001 |  | 4405 | 1∙17 (1∙09, 1∙25) | <0∙001 |
|  | **-1 to 1 SD** | 35630 | 1∙10 (1∙07, 1∙14) | <0∙001 |  | 30422 | 1∙10 (1∙06, 1∙14) | <0∙001 |
|  | **1 to 2 SD** | 7806 | 1∙10 (1∙04, 1∙16) | <0∙001 |  | 7232 | 1∙11 (1∙05, 1∙17) | <0∙001 |
|  | **>=2 SD** | 1759 | 1∙22 (1∙10, 1∙35) | <0∙001 |  | 1671 | 1∙20 (1∙09, 1∙34) | 0∙001 |
| **>=40 weeks** | **<-2 SD** | 1239 | 1∙06 (0∙94, 1∙21) | 0∙326 |  | 1068 | 1∙10 (0∙97, 1∙26) | 0∙147 |
|  | **-2 to -1 SD** | 7303 | 1∙05 (0∙99, 1∙11) | 0∙093 |  | 6394 | 1∙05 (0∙99, 1∙11) | 0∙137 |
|  | **-1 to 1 SD** | 46339 | REF |  |  | 43077 | REF |  |
|  | **1 to 2 SD** | 11092 | 1∙03 (0∙99, 1∙08) | 0∙155 |  | 10797 | 1∙04 (0∙99, 1∙09) | 0∙134 |
|  | **>=2 SD** | 2435 | 1∙07 (0∙97, 1∙17) | 0∙176 |  | 2400 | 1∙07 (0∙97, 1∙17) | 0∙156 |
|  |  |  | **TOTAL EUROPE** |  |  |  | **NORTH-WEST EUROPE** |  |
|  |  |  | **(n = 129,813)** |  |  |  | **(n = 117,352)** |  |
|  |  | **n** | **Odds Ratio (95% CI)** | **p-value** |  | **n** | **Odds Ratio (95% CI)** | **p-value** |
| **Gestational age** | **Weight gain** |  |  |  |  |  |  |  |
| **<32 weeks** | **<500 grams** | 87 | 1∙89 (1∙21, 2∙96) | 0∙005 |  | 70 | 1∙82 (1∙10, 3∙00) | 0∙020 |
|  | **500-600 grams** | 178 | 2∙35 (1∙73, 3∙20) | <0∙001 |  | 142 | 2∙59 (1∙84, 3∙64) | <0∙001 |
|  | **600-700 grams** | 163 | 1∙78 (1∙29, 2∙46) | <0∙001 |  | 144 | 2∙08 (1∙48, 2∙92) | <0∙001 |
|  | **>=700 grams** | 81 | 3∙27 (2∙06, 5∙19) | <0∙001 |  | 75 | 3∙27 (2∙03, 5∙27) | <0∙001 |
| **32-36 weeks** | **<500 grams** | 314 | 1∙21 (0∙94, 1∙54) | 0∙136 |  | 257 | 1∙24 (0∙94, 1∙62) | 0∙127 |
|  | **500-600 grams** | 839 | 1∙39 (1∙19, 1∙61) | <0∙001 |  | 717 | 1∙41 (1∙20, 1∙65) | <0∙001 |
|  | **600-700 grams** | 765 | 1∙62 (1∙39, 1∙88) | <0∙001 |  | 673 | 1∙61 (1∙37, 1∙90) | <0∙001 |
|  | **>=700 grams** | 437 | 2∙04 (1∙68, 2∙49) | <0∙001 |  | 358 | 2∙08 (1∙68, 2∙59) | <0∙001 |
| **36-40 weeks** | **<500 grams** | 12107 | 0∙96 (0∙92, 1∙02) | 0∙166 |  | 10314 | 0∙97 (0∙92, 1∙02) | 0∙245 |
|  | **500-600 grams** | 16593 | 1∙07 (1∙02, 1∙11) | 0∙006 |  | 14571 | 1∙06 (1∙01, 1∙11) | 0∙014 |
|  | **600-700 grams** | 9199 | 1∙27 (1∙20, 1∙33) | <0∙001 |  | 8084 | 1∙27 (1∙20, 1∙34) | <0∙001 |
|  | **>=700 grams** | 4069 | 1∙51 (1∙40, 1∙63) | <0∙001 |  | 3167 | 1∙53 (1∙41, 1∙66) | <0∙001 |
| **>=40 weeks** | **<500 grams** | 20184 | 0∙88 (0∙84, 0∙92) | <0∙001 |  | 18489 | 0∙88 (0∙84, 0∙92) | <0∙001 |
|  | **500-600 grams** | 22337 | REF |  |  | 21149 |  |  |
|  | **600-700 grams** | 10284 | 1∙15 (1∙10, 1∙22) | <0∙001 |  | 9745 | 1∙16 (1∙10, 1∙22) | <0∙001 |
|  | **>=700 grams** | 3721 | 1∙30 (1∙20, 1∙40) | <0∙001 |  | 3377 | 1∙31 (1∙21, 1∙41) | <0∙001 |
|  |  |  |  |  |  |  |  |  |
| **Birth weight** | **Weight gain** |  |  |  |  |  |  |  |
| **<-2 SD** | **<500 grams** | 550 | 0∙92 (0∙76, 1∙12) | 0∙410 |  | 433 | 0∙96 (0∙77, 1∙19) | 0∙693 |
|  | **500-600 grams** | 804 | 1∙09 (0∙94, 1∙28) | 0∙251 |  | 707 | 1∙11 (0∙94, 1∙31) | 0∙210 |
|  | **600-700 grams** | 462 | 1∙18 (0∙97, 1∙43) | 0∙106 |  | 424 | 1∙17 (0∙95, 1∙44) | 0∙137 |
|  | **>=700 grams** | 210 | 1∙05 (0∙79, 1∙40) | 0∙743 |  | 177 | 1∙08 (0∙79,1∙48) | 0∙628 |
|  |  |  | **TOTAL EUROPE** |  |  |  | **NORTH-WEST EUROPE** |  |
|  |  |  | **(n = 129,813)** |  |  |  | **(n = 117,352)** |  |
|  |  | **n** | **Odds Ratio (95% CI)** | **p-value** |  | **n** | **Odds Ratio (95% CI)** | **p-value** |
| **Birth weight (continued)** | **Weight gain (continued)** |  |  |  |  |  |  |  |
| **-2 to -1 SD** | **<500 grams** | 3128 | 0∙92 (0∙84, 1∙00) | 0∙055 |  | 2507 | 0∙92 (0∙84, 1∙01) | 0∙098 |
|  | **500-600 grams** | 4222 | 1∙07 (1∙00, 1∙15) | 0∙051 |  | 3676 | 1∙09 (1∙01, 1∙18) | 0∙032 |
|  | **600-700 grams** | 2287 | 1∙17 (1∙06, 1∙28) | 0∙001 |  | 2014 | 1∙15 (1∙04, 1∙27) | 0∙006 |
|  | **>=700 grams** | 962 | 1∙43 (1∙25, 1∙64) | <0∙001 |  | 800 | 1∙42 (1∙23, 1∙65) | <0∙001 |
| **-1 to 1 SD** | **<500 grams** | 21459 | 0∙88 (0∙84, 0∙92) | <0∙001 |  | 18951 | 0∙88 (0∙84, 0∙92) | <0∙001 |
|  | **500-600 grams** | 27207 | REF |  |  | 24781 | REF |  |
|  | **600-700 grams** | 13873 | 1∙19 (1∙14, 1∙24) | <0∙001 |  | 12589 | 1∙20 (1∙15, 1∙26) | <0∙001 |
|  | **>=700 grams** | 5650 | 1∙37 (1∙28, 1∙46) | <0∙001 |  | 4665 | 1∙39 (1∙30, 1∙48) | <0∙001 |
| **1 to 2 SD** | **<500 grams** | 5553 | 0∙93 (0∙87, 1∙00) | 0∙048 |  | 5295 | 0∙94 (0∙88, 1∙01) | 0∙089 |
|  | **500-600 grams** | 5930 | 1∙00 (0∙94, 1∙06) | 0∙960 |  | 5681 | 1∙01 (0∙95, 1∙08) | 0∙687 |
|  | **600-700 grams** | 2975 | 1∙20 (1∙11, 1∙31) | <0∙001 |  | 2831 | 1∙21 (1∙11, 1∙31) | <0∙001 |
|  | **>=700 grams** | 1189 | 1∙52 (1∙34, 1∙71) | <0∙001 |  | 1056 | 1∙52 (1∙33, 1∙72) | <0∙001 |
| **>=2 SD** | **<500 grams** | 1516 | 0∙93 (0∙83, 1∙05) | 0∙243 |  | 1469 | 0∙93 (0∙82, 1∙05) | 0∙248 |
|  | **500-600 grams** | 1228 | 1∙11 (0∙98, 1∙25) | 0∙115 |  | 1195 | 1∙10 (0∙97, 1∙25) | 0∙153 |
|  | **600-700 grams** | 563 | 1∙29 (1∙08, 1∙54) | 0∙005 |  | 543 | 1∙30 (1∙08, 1∙56) | 0∙005 |
|  | **>=700 grams** | 209 | 1∙92 (1∙45, 2∙55) | <0∙001 |  | 192 | 2∙01 (1∙50, 2∙69) | <0∙001 |

Values are odds ratios (95% confidence interval) from multi-level regression analysis. Values reflect the odds of wheezing compared with children born at term with a normal birth weight for gestational age, born at term with moderate infant weight gain, and born with a normal birth weight for gestational age and moderate infant weight gain. Models are adjusted for maternal educational level, smoking during pregnancy, history of asthma, and smoking during infancy, and for child’s sex, siblings, and attending day care. Total analysis includes all cohorts, North-West includes cohorts in Northern and Western Europe according to the UN definition (http://unstats∙un∙org/unsd/methods/m49/m49regin∙htm#europe, assessed 31 May 2013): Denmark, France, Germany, Ireland, Netherlands, Norway, Sweden, Switzerland, and United Kingdom.

**Table E9. Associations of gestational age, birth weight, and infant weight gain with school-age asthma in all countries (as presented in Figure 2 in the main manuscript), and in North-West European cohorts only**

|  |  |  | **TOTAL EUROPE** |  |  |  | **NORTH-WEST EUROPE** |  |
| --- | --- | --- | --- | --- | --- | --- | --- | --- |
|  |  |  | **(n = 93,124)** |  |  |  | **(n = 83,890)** |  |
|  |  | **n** | **Odds Ratio (95% CI)** | **p-value** |  | **n** | **Odds Ratio (95% CI)** | **p-value** |
| **Gestational age** | **Birth weight** |  |  |  |  |  |  |  |
| **<32 weeks** | **<-2 SD** | 11 | NA | NA |  | 10 | NA | NA |
|  | **-2 to -1 SD** | 55 | 1∙59 (0∙77, 3∙30) | 0∙210 |  | 45 | 1∙73 (0∙82, 3∙63) | 0∙148 |
|  | **-1 to 1 SD** | 247 | 1∙85 (1∙33, 2∙58) | <0∙001 |  | 199 | 1∙95 (1∙39, 2∙74) | 0∙000 |
|  | **1 to 2 SD** | 33 | 3∙47 (1∙65, 7∙31) | 0∙001 |  | 27 | 3∙13 (1∙38, 7∙09) | 0∙006 |
|  | **>=2 SD** | 10 | 2∙63 (0∙53, 13∙13) | 0∙238 |  | 5 | 1∙69 (0∙18, 15∙75) | 0∙644 |
| **32-36 weeks** | **<-2 SD** | 82 | 1∙39 (0∙76, 2∙56) | 0∙286 |  | 69 | 1∙23 (0∙64, 2∙37) | 0∙537 |
|  | **-2 to -1 SD** | 243 | 2∙42 (1∙77, 3∙30) | <0∙001 |  | 196 | 2∙49 (1∙80, 3∙45) | 0∙000 |
|  | **-1 to 1 SD** | 1398 | 1∙78 (1∙55, 2∙06) | <0∙001 |  | 1148 | 1∙79 (1∙54, 2∙08) | 0∙000 |
|  | **1 to 2 SD** | 256 | 1∙83 (1∙32, 2∙53) | <0∙001 |  | 220 | 1∙90 (1∙36, 2∙66) | 0∙000 |
|  | **>=2 SD** | 73 | 1∙68 (0∙90, 3∙12) | 0∙103 |  | 63 | 1∙47 (0∙76, 2∙88) | 0∙256 |
| **36-40 weeks** | **<-2 SD** | 789 | 1∙31 (1∙07, 1∙61) | 0∙009 |  | 657 | 1∙35 (1∙10, 1∙67) | 0∙005 |
|  | **-2 to -1 SD** | 3863 | 1∙29 (1∙17, 1∙43) | <0∙001 |  | 3087 | 1∙33 (1∙20, 1∙48) | 0∙000 |
|  | **-1 to 1 SD** | 25833 | 1∙17 (1∙11, 1∙23) | <0∙001 |  | 21935 | 1∙18 (1∙12, 1∙24) | 0∙000 |
|  | **1 to 2 SD** | 5723 | 1∙25 (1∙15, 1∙36) | <0∙001 |  | 5263 | 1∙27 (1∙16, 1∙38) | 0∙000 |
|  | **>=2 SD** | 1228 | 1∙33 (1∙13, 1∙57) | 0∙001 |  | 1151 | 1∙32 (1∙12, 1∙57) | 0∙001 |
| **>=40 weeks** | **<-2 SD** | 861 | 1∙31 (1∙08, 1∙58) | 0∙006 |  | 751 | 1∙33 (1∙10, 1∙62) | 0∙004 |
|  | **-2 to -1 SD** | 5150 | 1∙11 (1∙02, 1∙22) | 0∙017 |  | 4551 | 1∙10 (1∙01, 1,21) | 0∙037 |
|  | **-1 to 1 SD** | 33932 | REF |  |  | 31552 | REF |  |
|  | **1 to 2 SD** | 8221 | 0∙99 (0∙92, 1∙07) | 0∙891 |  | 7986 | 1∙00 (0∙92, 1∙08) | 0∙976 |
|  | **>=2 SD** | 1787 | 0∙98 (0∙84, 1∙14) | 0∙765 |  | 1761 | 0∙97 (0∙83, 1∙14) | 0∙730 |
|  |  |  | **TOTAL EUROPE** |  |  |  | **NORTH-WEST EUROPE** |  |
|  |  |  | **(n = 93,124)** |  |  |  | **(n = 83,890)** |  |
|  |  | **n** | **Odds Ratio (95% CI)** | **p-value** |  | **n** | **Odds Ratio (95% CI)** | **p-value** |
| **Gestational age** | **Weight gain** |  |  |  |  |  |  |  |
| **<32 weeks** | **<500 grams** | 55 | 1∙10 (0∙46, 2∙63) | 0∙825 |  | 41 | 1∙23 (0∙51, 2∙98) | 0∙639 |
|  | **500-600 grams** | 113 | 2∙17 (1∙32, 3∙55) | 0∙002 |  | 80 | 2∙19 (1∙29, 3∙69) | 0∙003 |
|  | **600-700 grams** | 114 | 1∙99 (1∙23, 3∙22) | 0∙005 |  | 98 | 2∙00 (1∙22, 3∙29) | 0∙006 |
|  | **>=700 grams** | 59 | 4∙47 (2∙58, 7∙76) | <0∙001 |  | 56 | 4∙34 (2∙48, 7,62) | 0∙000 |
| **32-36 weeks** | **<500 grams** | 183 | 1∙60 (1∙05, 2∙43) | 0∙029 |  | 155 | 1∙49 (0∙96, 2∙32) | 0∙079 |
|  | **500-600 grams** | 511 | 1∙84 (1∙44, 2∙35) | <0∙001 |  | 421 | 1∙84 (1∙43, 2∙38) | 0∙000 |
|  | **600-700 grams** | 472 | 1∙70 (1∙33, 2∙18) | <0∙001 |  | 404 | 1∙71 (1∙33, 2∙20) | 0∙000 |
|  | **>=700 grams** | 239 | 2∙06 (1∙51, 2∙82) | <0∙001 |  | 213 | 2∙17 (1∙58, 2∙98) | 0∙000 |
| **36-40 weeks** | **<500 grams** | 7339 | 1∙12 (1∙02, 1∙23) | 0∙020 |  | 6373 | 1∙13 (1∙03, 1∙24) | 0∙012 |
|  | **500-600 grams** | 10822 | 1∙19 (1∙09, 1∙28) | <0∙001 |  | 9408 | 1∙19 (1∙10, 1∙29) | 0∙000 |
|  | **600-700 grams** | 6036 | 1∙32 (1∙20, 1∙44) | <0∙001 |  | 5285 | 1∙33 (1∙21, 1∙45) | 0∙000 |
|  | **>=700 grams** | 2301 | 1∙44 (1∙27, 1∙62) | <0∙001 |  | 2085 | 1∙46 (1∙20, 1∙65) | 0∙000 |
| **>=40 weeks** | **<500 grams** | 12357 | 0∙95 (0∙88, 1∙03) | 0∙207 |  | 11798 | 0∙96 (0∙99, 1∙04) | 0∙271 |
|  | **500-600 grams** | 14478 | REF |  |  | 13783 | REF |  |
|  | **600-700 grams** | 6697 | 1∙11 (1∙02, 1∙21) | 0∙020 |  | 6378 | 1∙12 (1∙03, 1∙23) | 0∙010 |
|  | **>=700 grams** | 2316 | 1∙22 (1∙08, 1∙38) | 0∙002 |  | 2233 | 1∙21 (1∙07, 1∙38) | 0∙002 |
|  |  |  |  |  |  |  |  |  |
| **Birth weight** | **Weight gain** |  |  |  |  |  |  |  |
| **<-2 SD** | **<500 grams** | 319 | 1∙16 (0∙83, 1∙63) | 0∙394 |  | 319 | 1∙16 (0∙83, 1∙63) | 0∙394 |
|  | **500-600 grams** | 501 | 1∙00 (0∙76, 1∙31) | 0∙973 |  | 501 | 1∙00 (0∙76, 1∙31) | 0∙973 |
|  | **600-700 grams** | 305 | 1∙55 (1∙16, 2∙08) | 0∙003 |  | 305 | 1∙55 (1∙16, 2∙08) | 0∙003 |
|  | **>=700 grams** | 123 | 1∙90 (1∙26, 2∙88) | 0∙002 |  | 123 | 1∙90 (1∙26∙ 2∙88) | 0∙002 |
|  |  |  | **TOTAL EUROPE** |  |  |  | **NORTH-WEST EUROPE** |  |
|  |  |  | **(n = 93,124)** |  |  |  | **(n = 83,890)** |  |
|  |  | **n** | **Odds Ratio (95% CI)** | **p-value** |  | **n** | **Odds Ratio (95% CI)** | **p-value** |
| **Birth weight (continued)** | **Weight gain (continued)** |  |  |  |  |  |  |  |
| **-2 to -1 SD** | **<500 grams** | 1834 | 0∙95 (0∙81, 1∙12) | 0∙532 |  | 1834 | 0∙95 (0∙81, 1∙12) | 0∙532 |
|  | **500-600 grams** | 2690 | 1∙06 (0∙93, 1∙20) | 0∙385 |  | 2690 | 1∙06 (0∙93, 1∙20) | 0∙385 |
|  | **600-700 grams** | 1489 | 1∙16 (1∙00, 1∙36) | 0∙052 |  | 1489 | 1∙16 (1∙00, 1∙36) | 0∙052 |
|  | **>=700 grams** | 550 | 1∙53 (1∙23, 1∙89) | <0∙001 |  | 550 | 1∙53 (1∙23, 1∙89) | 0∙000 |
| **-1 to 1 SD** | **<500 grams** | 13194 | 0∙94 (0∙87, 1∙01) | 0∙086 |  | 13194 | 0∙94 (0∙87, 1∙01) | 0∙086 |
|  | **500-600 grams** | 17731 | REF |  |  | 17731 | REF |  |
|  | **600-700 grams** | 9074 | 1∙09 (1∙01, 1∙18) | 0∙025 |  | 9074 | 1∙09 (1∙01, 1∙18) | 0∙025 |
|  | **>=700 grams** | 3328 | 1∙21 (1∙09, 1∙34) | <0∙001 |  | 3328 | 1∙21 (1∙09, 1∙34) | 0∙000 |
| **1 to2 SD** | **<500 grams** | 3386 | 0∙95 (0∙84, 1∙07) | 0∙407 |  | 3386 | 0∙95 (0∙84, 1∙07) | 0∙407 |
|  | **500-600 grams** | 3887 | 1∙02 (0∙91, 1∙14) | 0∙722 |  | 3887 | 1∙02 (0∙01, 1∙14) | 0∙722 |
|  | **600-700 grams** | 1922 | 1∙16 (1∙01, 1∙33) | 0∙035 |  | 1922 | 1∙16 (1∙01, 1∙33) | 0∙035 |
|  | **>=700 grams** | 751 | 1∙31 (1∙08, 1∙59) | 0∙007 |  | 751 | 1∙31 (1∙08, 1∙59) | 0∙007 |
| **>=2 SD** | **<500 grams** | 918 | 0∙89 (0∙71, 1∙11) | 0∙307 |  | 918 | 0∙89 (0∙71, 1∙11) | 0∙307 |
|  | **500-600 grams** | 760 | 1∙08 (0∙86, 1∙35) | 0∙503 |  | 760 | 1∙08 (0∙86, 1∙35) | 0∙503 |
|  | **600-700 grams** | 374 | 1∙68 (1∙29, 2∙19) | <0∙001 |  | 374 | 1∙68 (1∙29, 2∙19) | 0∙000 |
|  | **>=700 grams** | 111 | 1∙11 (0∙67, 1∙85) | 0∙684 |  | 111 | 1∙11 (0∙67, 1∙85) | 0∙684 |

Values are odds ratios (95% confidence interval) from multi-level regression analysis. Values reflect the odds of asthma compared with children born at term with a normal birth weight for gestational age, born at term with moderate infant weight gain, and born with a normal birth weight for gestational age and moderate infant weight gain. Models are adjusted for maternal educational level, smoking during pregnancy, history of asthma, and smoking during infancy, and for child’s sex, siblings, and attending day care. Total analysis includes all cohorts, North-West includes cohorts in Northern and Western Europe according to the UN definition (http://unstats∙un∙org/unsd/methods/m49/m49regin∙htm#europe, assessed 31 May 2013): Denmark, France, Germany, Ireland, Netherlands, Norway, Sweden, Switzerland, and United Kingdom.

**Table E10. Sensitivity analyses of associations of gestational age at birth and birth weight with preschool wheezing**

|  | **Wheezing (95% confidence interval)** | | |
| --- | --- | --- | --- |
|  | **Model as presented in Figure 1** | **Model with only records of early growth data** | **Models with only ISAAC based wheezing data** |
| **Gestational age at birth** |  |  |  |
| <28 | 3.86 (2.66, 5.60) | 3.63 (2.47, 5.34) | 4.29 (2.79, 6.61) |
| 28-29.9 | 2.59 (1.88, 3.55) | 2.47 (1.79, 3.43) | 2.39 (1.67, 3.41) |
| 30-31.9 | 1.77 (1.40, 2.22) | 1.80 (1.42, 2.29) | 1.75 (1.35, 2.25) |
| 32-33.9 | 1.76 (1.51, 2.05) | 1.69 (1.44, 1.99) | 1.67 (1.41, 1.98) |
| 34-35.9 | 1.43 (1.30, 1.58) | 1.43 (1.29, 1.59) | 1.38 (1.24, 1.53) |
| 36-37.9 | 1.22 (1.16, 1.28) | 1.22 (1.16, 1.29) | 1.23 (1.16, 1.30) |
| 38-39.9 | 1.08 (1.05, 1.11) | 1.08 (1.05, 1.11) | 1.08 (1.05, 1.11) |
| 40-41.9 | *Reference* | *Reference* | *Reference* |
| =>42 | 1.03 (0.98, 1.08) | 1.02 (0.97, 1.07) | 1.02 (0.96, 1.07) |
|  |  |  |  |
| **Birth weight SDS** |  |  |  |
| <-4 | 1.26 (0.59, 2.69) | 1.70 (0.68, 4.23) | 1.64 (0.63, 4.29) |
| -4 - -3.01 | 1.25 (0.96, 1.62) | 1.25 (0.95, 1.66) | 1.29 (0.96, 1.73) |
| -3 - -2.01 | 1.00 (0.91, 1.10) | 1.01 (0.91, 1.11) | 1.04 (0.94, 1.16) |
| -2 - -1.01 | 1.03 (0.99, 1.08) | 1.02 (0.97, 1.06) | 1.04 (0.99, 1.09) |
| -1 - -0.01 | 0.97 (0.94, 1.00) | 0.97 (0.94, 1.00) | 0.98 (0.95, 1.01) |
| 0-0.99 | *Reference* | *Reference* | *Reference* |
| 1-1.99 | 1.01 (0.97, 1.04) | 1.00 (0.96, 1.04) | 1.00 (0.96, 1.04) |
| 2-2.99 | 1.08 (1.01, 1.16) | 1.11 (1.03, 1.20) | 1.07 (0.99, 1.16) |
| 3-3.99 | 0.94 (0.76, 1.15) | 0.91 (0.73, 1.13) | 0.95 (0.77, 1.18) |
| =>4 | 0.98 (0.56, 1.72) | 0.96 (0.53, 1.75) | 1.04 (0.58, 1.85) |

Values are odds ratios (95% confidence interval) from random effect multi-level models for the associations of gestational age at birth and gestational age adjusted birth weight (birth weight SDS) with preschool wheezing. Models were adjusted for maternal educational level, smoking during pregnancy, history of asthma, and smoking during infancy, and for child’s sex, siblings, and attending day care. Gestational age was additionally adjusted for birth weight.

**Table E11. Sensitivity analyses of associations of gestational age at birth and birth weight with asthma**

|  | **Wheezing (95% confidence interval)** | | |
| --- | --- | --- | --- |
|  | **Model as presented in Figure 1** | **Model with only records of early growth data** | **Models with only ISAAC based asthma data** |
| **Gestational age at birth** |  |  |  |
| <28 | 2.83 (1.76, 4.57) | 2.96 (1.83, 4.81) | 3.44 (2.06, 5.73) |
| 28-29.9 | 1.44 (0.87, 2.38) | 1.51 (0.91, 2.51) | 1.57 (0.92, 2.69) |
| 30-31.9 | 1.88 (1.37, 2.59) | 1.86 (1.35, 2.57) | 2.14 (1.52, 3.01) |
| 32-33.9 | 2.30 (1.85, 2.86) | 2.31 (1.84, 2.90) | 2.43 (1.92, 3.08) |
| 34-35.9 | 1.55 (1.34, 1.81) | 1.57 (1.34, 1.83) | 1.64 (1.39, 1.93) |
| 36-37.9 | 1.36 (1.25, 1.48) | 1.36 (1.25, 1.48) | 1.43 (1.30, 1.56) |
| 38-39.9 | 1.13 (1.08, 1.19) | 1.13 (1.08, 1.19) | 1.15 (1.10, 1.21) |
| 40-41.9 | *Reference* | *Reference* | *Reference* |
| =>42 | 0.98 (0.90, 1.06) | 0.97 (0.89, 1.06) | 0.96 (0.88, 1.05) |
|  |  |  |  |
| **Birth weight SDS** |  |  |  |
| <-4 | 2.06 (0.73, 5.89) | 2.54 (0.86, 7.48) | 1.05 (0.24, 4.71) |
| -4 - -3.01 | 1.55 (1.07, 2.23) | 1.59 (1.10, 2.32) | 1.66 (1.10, 2.50) |
| -3 - -2.01 | 1.12 (0.97, 1.30) | 1.08 (0.93, 1.26) | 1.07 (0.91, 1.27) |
| -2 - -1.01 | 1.10 (1.03, 1.18) | 1.11 (1.03, 1.19) | 1.11 (1.03, 1.20) |
| -1 - -0.01 | 0.97 (0.93, 1.02) | 0.97 (0.92, 1.02) | 0.96 (0.91, 1.02) |
| 0-0.99 | *Reference* | *Reference* | *Reference* |
| 1-1.99 | 1.02 (0.96, 1.08) | 1.00 (0.94, 1.07) | 1.03 (0.97, 1.10) |
| 2-2.99 | 1.03 (0.92, 1.16) | 1.06 (0.93, 1.19) | 1.01 (0.89, 1.14) |
| 3-3.99 | 1.04 (0.75, 1.46) | 1.05 (0.74, 1.47) | 1.08 (0.76, 1.53) |
| =>4 | 1.12 (0.46, 2.70) | 1.18 (0.49, 2.86) | 0.82 (0.29, 2.34) |

Values are odds ratios (95% confidence interval) from random effect multi-level models for the associations of gestational age at birth and gestational age adjusted birth weight (birth weight SDS) with school age asthma. Models were adjusted for maternal educational level, smoking during pregnancy, history of asthma, and smoking during infancy, and for child’s sex, siblings, and attending day care. Gestational age was additionally adjusted for birth weight.
